# Hobson’s choice or a horned dilemma: a grounded theory on adherence to adjuvant endocrine therapy verified with breast cancer survivors

**DOI:** 10.1007/s00520-022-07435-2

**Published:** 2022-11-07

**Authors:** Othman AlOmeir, Nilesh Patel, Parastou Donyai

**Affiliations:** 1 Department of Pharmacy, University of Reading, Whiteknights, Reading, PO Box 226, Berkshire, RG6 6AP UK; 2 Department of Pharmacy Practice, College of Pharmacy, Shaqra University, Dawadmi, Saudi Arabia

**Keywords:** Grounded theory, Adherence, Medication, Breast cancer, Hormone therapy, Qualitative research

## Abstract

**Purpose:**

A literature review and meta-synthesis of qualitative research had enabled us to develop a grounded theory explaining the difficulties breast cancer survivors face with the initial decision to accept long-term endocrine therapy, and the everyday challenges of continuing or deciding to stop treatment early. Our objective was to interview a cohort of women in a UK setting to corroborate and complete the grounded theory with the end users’ primary involvement.

**Methods:**

A semi-structured interview schedule was written based on the existing grounded theory. Fourteen women with a history of hormone-positive breast cancer were recruited and interviewed. The audio-recorded interviews were transcribed and analysed against the existing grounded theory.

**Results:**

The findings were compatible with the core theory ‘Hobson’s choice or a horned dilemma’ and its constituent categories previously developed, with additional concepts identified and added to our paradigm models. Importantly, we found that some women who started with a strong sense of commitment to their treatment changed their mind as they experienced the medication side effects over time, impacting on their persistence with long-term endocrine therapy.

**Conclusion:**

The findings indicate an opportunity for health providers to intervene and influence women’s waning perceptions of the necessity of their treatment, for example upon experiencing the side effects. Interventions could involve the provision of side effect management strategies via accessible resources.

**Supplementary information:**

The online version contains supplementary material available at 10.1007/s00520-022-07435-2.

## Introduction

According to the World Health Organisation, there were 2.3 million women diagnosed with breast cancer globally in 2020 with 7.8 million women having been living with breast cancer in the prior 5 years, making this the most prevalent cancer worldwide [1]. Many women are treated successfully for this condition due to advancements that include adjuvant treatment with endocrine therapies such as tamoxifen and aromatase inhibitors for hormone receptor (HR) positive cancers. These medicines, taken orally for 5–10 years, significantly reduce the chances of recurrence of HR positive cases [2]. However, because tamoxifen and aromatase inhibitors target and diminish oestrogen activity in the body, they also create a range of side effects related directly to their pharmacological mode of action. These include vasomotor symptoms (hot flushes, night sweats), menstrual abnormalities/irregularities, vaginal discharge, and vaginal dryness [3]. Research has shown a direct relationship between experiencing these side effects and women’s non-adherence and/or non-persistence with their oral adjuvant therapy [4]. There are also other correlates of adherence and persistence [5], some of which are not modifiable, such as cytochrome P450 2D6 (a liver enzyme involved in drug metabolism) activity, but some of which are, such as drug costs, and follow-up care provision (general practitioner versus oncologist) [4].

By completing a grounded theory meta-synthesis of the published literature, we created a theory to explain the challenges of taking adjuvant endocrine therapy in breast cancer and the resilience needed to continue, as briefly outlined here [6]. Our core theory was that women’s decision to take adjuvant endocrine therapy is, to them, not seen as a choice at all, or when there is a choice, it is between two equally bad options. At the start of their treatment journey, the only choice given to women is to start adjuvant endocrine therapy, then as the treatment progresses and women experience numerous medication side effects, they feel they must either tolerate these adverse effects or stop the treatment altogether and risk losing the protective benefit of their medication. Eventually, some women feel empowered to stop their medication altogether prioritising quality of life over longevity. These detailed categories were constructed within ‘paradigm models’ which encompassed the specific context, causal conditions, actions/interactions, and consequences for women, as well as the mediating factors which influenced the different actions and interactions [6]. Our theory explained why adherence decreases over time [7], and importantly that the decision to cease treatment early was an active choice made with a credible rationale. One of the limitations of our meta-synthesis, however, had been our lack of access to original interview transcripts, our analysis instead built on the quotes extracted by the original authors and their respective interpretations. Thus, our objective here was to interview a cohort of women in a UK setting to corroborate and complete the grounded theory and its constituent categories against primary data to produce an updated and validated explanatory model of adjuvant endocrine therapy and medication taking in breast cancer survivorship.

## Material and methods

### Design and sampling

The prescribing of adjuvant endocrine therapy for breast cancer in the UK is usually initiated within secondary care and continued in general practice, with at least yearly hospital reviews [8]. The population of interest was women diagnosed with breast cancer who were/had been receiving a prescription for an oral endocrine medication (tamoxifen or aromatase inhibitors; anastrozole, exemestane, letrozole) for the long-term management of breast cancer. In our interviews, we used open-ended questions relating to the broad stages of treatment identified in the grounded theory, namely starting, continuing, and stopping treatment, as appropriate. In line with theoretical sampling, we continued recruitment to verify or refute existing themes or for gathering additional ideas to illuminate and define the properties, boundaries, and relevance of the categories through focussed questions [9]. Recruitment was via the university’s staff email list (*N* = 9) and through local breast cancer support groups (*N* = 5) identified from the website of the MacMillan Cancer Network. We excluded women in an acute state of illness, those unable to consent due to language barriers, and women who had not received adjuvant endocrine therapy for their breast cancer.

**Fig. 1:**
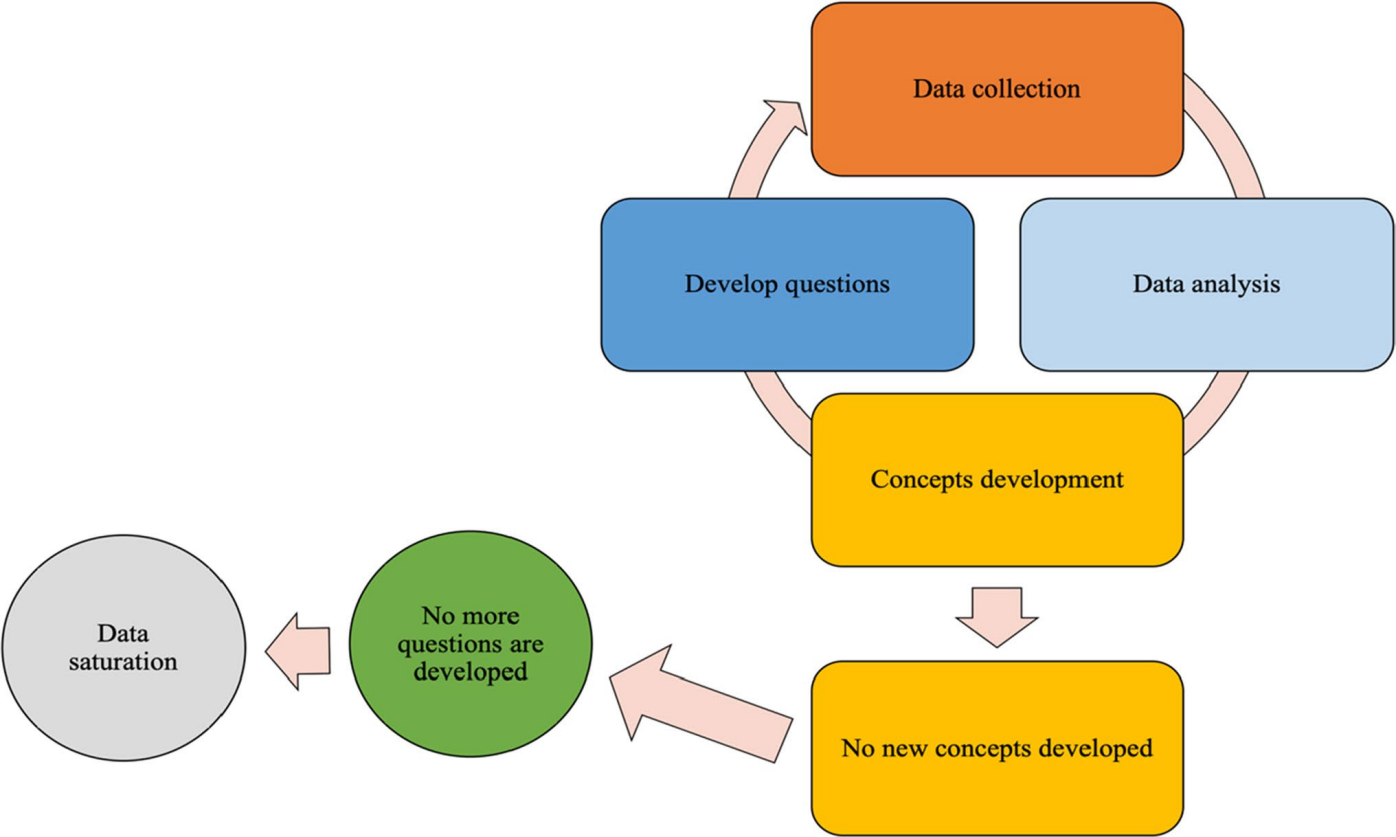
The process of data collection and identifying data saturation

One author (OA) carried out in-depth semi-structured interviews either face to face (*N* = 11) or using the telephone (*N* = 3), making contemporaneous field notes. Written consent was obtained from each participant prior to interview, including a separate signature to consent to audio-recording of the interview, and to being contacted again to review the interview transcript in due course. Each participant received an online Amazon voucher (£20) after the interview. The interviews were conducted in a private room on the university campus. There were no repeat interviews and each interview lasted 40–85 min (average 59 min). We continued to collect data until the properties of our theoretical categories were saturated meaning fresh data no longer sparked new theoretical insights or revealed new properties of the core theoretical categories [8] (see Fig. 1).

### Setting and participants

Participants were given a code based on the sequential order of their recruitment, as shown in Table 1.

**Table 1:** Participant characteristics and detail of interviews

Participant code	Age group	Medication history	Duration taking the treatment	Interview duration	Interview mode
Interview 1	50–59	Anastrozole	Stopped after 5 years	45:21	Face to face interview
Interview 2	60–69	Anastrozole	16 years on the treatment and still continuing	42:53	Face to face interview
Interview 3	50–59	Tamoxifen then switched to exemestane	5 years on the treatment and still continuing	55:53	Face to face interview
Interview 4	60–69	Anastrozole	4 years on the treatment and still continuing	50:48	Face to face interview
Interview 5	50–59	Tamoxifen then switched to letrozole	Stopped the treatment after 10 years — 5 years on each treatment	41:07	Face to face interview
Interview 6	50–59	Anastrozole	4 years on the treatment and still continuing	46:14	Face to face interview
Interview 7	50–59	Tamoxifen	8 years on the treatment and still continuing	53:18	Face to face interview
Interview 8	40–49	Tamoxifen	2 months on the treatment and still continuing	39:38	Face to face interview
Interview 9	60–69	Anastrozole	6 years on the treatment and still continuing	1:12:28	Face to face interview
Interview 10	50–59	Anastrozole	18 months on the treatment and still continuing	52:51	Face to face interview
Interview 11	50–59	Anastrozole	5 years on the treatment and still continuing	1:21:35	Phone interview
interview 12	50–59	Tamoxifen then switched to letrozole	3rd year on the treatment	1:04:37	Phone interview
Interview 13	> 70	Tamoxifen then switched to anastrozole	Stopped the treatment after 8 years	1:24:31	Phone interview
Interview 14	> 70	Tamoxifen then switched to letrozole	Stopped after 5 years and refused to continue further	1:22:49	Face to face interview

### Data analysis

The interviews were transcribed verbatim with the help of a Transcription company, password-protected and pseudo-anonymised so that they contained no identifying information. The first author (OA) ensured data integrity in consultation with the senior author (PD). The Strauss and Corbin methodology was used for the analysis [10]. The software programme NVivo (v.11) was used to facilitate the analysis of interview responses. Because of the existing grounded theory developed via the meta-synthesis, coding involved a combination of both inductive and deductive analysis, such that after exploring the data using the preconceived categories, the analysis continued to isolate new concepts where possible.

**Fig. 2:**
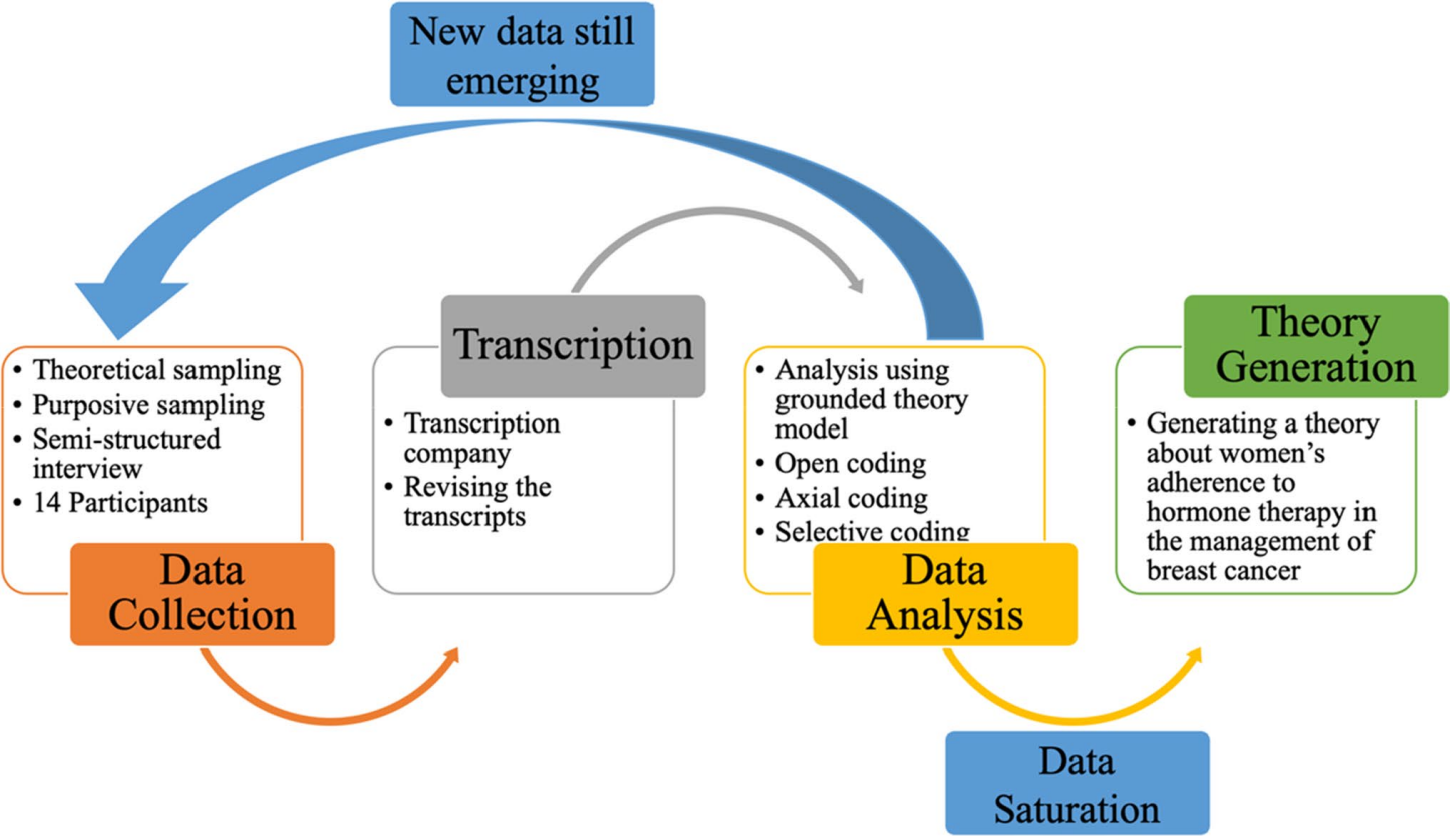
The process of data analysis

The analytical process began with close reading of the transcripts. Data was deconstructed, reorganised, and coded at multiple levels, with analysis moving from one level of abstraction to another, using coding and constant comparison, within and between interviews. Data were coded by OA in close consultation with PD at open, axial, and selective levels to develop the theoretical categories. Using the paradigm model [10], *causal conditions*, *actions/interactions*, and *consequences* were identified for each category during axial coding. Selective coding in this instance involved checking that the new codes and categories were still compatible with the existing core category developed via the meta-synthesis (see Fig. 2).

### Researcher characteristics and reflexivity

The research topic was selected for investigation by the researchers, all pharmacists, who believed it was valuable to check their novel grounded theory with end users. OA, a Saudi male pharmacist, and doctoral student conducted the interviews, while PD provided guidance and supported the analyses. OA undertook specific training during his PhD according to an annual Learning Needs Analysis. PD is a female pharmacist academic and a psychologist with a PhD who was able to bridge the clinical (endocrine therapy) and investigative (social psychology using grounded theory) domains during analysis. A second supervisor, NP, who was a male pharmacist academic with a PhD provided further contextual support and is co-author of this paper.

### Ethical considerations

This study was reviewed by the university’s Research Ethics Committee and received a favourable opinion on 18/1/18 (study reference UREC 17/51). We alerted the university ethics committee through protocol amendments and received supplementary ethical approvals when any changes were made to the original protocol.

## Results

The results of this study were compatible with the core theory and its constituent categories developed through our grounded theory literature review. However, we unearthed additional concepts, allowing us to update each of the three paradigm models as follows.

### Guided by the doctors: accepting the long-term prescription

Like the findings of our meta-synthesis, the participants in this study spoke about the transition to adjuvant endocrine therapy, the process of deciding whether to start this new treatment, their hopes and concerns, the information provided to them, their sense of vulnerability, and the support they received. A summary of the quotes corresponding to these themes is provided in the Appendix (Table S1). Additionally, we identified new themes or dimensions of existing themes through our interviews, enabling us to update the existing paradigm model. First, in terms of causal conditions, it was not only the lack of information or too much information that was an issue but also some women’s understanding of the information was problematic, highlighting health literacy as an important consideration at this stage. Second, again in terms of causal conditions, in addition to concerns about the necessity, efficacy, safety, and mechanism of action of their treatment, women can also question the suitability of their new prescription, especially if unclear about whether they were menopausal at the treatment’s start, which would affect the choice of treatment given to them. Third, an additional mediating factors was identified, which influenced the different actions and interactions, and this was the support women received from friends and family members during the initial prescribing process. Thus, for example, support from others helped women to understand the information better or process the situation more clearly. The updated paradigm model is shown in Table 2.

**Table 2:** The amended paradigm model for the category ‘Guided by the doctors: accepting the long-term prescription’

Context: Completing the acute stage of treatment for breast cancer *The care of women being treated for breast cancer, in the UK guided by the National Institute for Health and Care Excellence (NICE), involves treating women with chemotherapy, radiotherapy, or surgery at the acute stage and with long-term treatment with adjuvant endocrine therapy given as appropriate*				
	Causal conditions	Actions/interactions	Consequences	
Trust in their healthcare provider	Transitioning into a new stage of breast cancer treatment Being overwhelmed by information provided all at once Fear of cancer recurrence Fear of possible side effects of the new treatment Lack of specific information and uncertainty about the medication (*necessity*, *efficacy*, *safety*, *mechanism of action*, and *type*) or lack of understanding of the provided information Feeling vulnerable The memory of difficult experiences during the initial stage of the treatment (*at a personal level*, *professional level*, or *emotional level*)	Having a consultation about the medication where it is prescribed Accepting or deferring the treatment (*dependent on e.g. trusting their healthcare provider advice*, *awareness of the necessity of the medication*, *the level of stability in family and social life*, *emotional stability and support*, *desire to continue living cancer free*, *co-morbidities*, and *need of normalcy*) Women taking care of themselves Women looking for information elsewhere (*through specialised websites*, *specialised forums*, or *from other patients*)	Going along with the prescription for endocrine therapy Delaying the treatment with endocrine therapy Transitioning into the long-term treatment phase with ease (*trusting the treatment and finding the necessary support*) Having difficulties transitioning into the long-term treatment phase Being well informed by receiving the correct information (*not looking for information in the wrong places and correcting misconceptions about the treatment*) Being wrongly informed about the medication (*side effects*, *mechanism of action*, *efficacy*, and *safety*) Fewer hospital visits (*less communication with healthcare providers*) **Guided by the doctors: accepting the long-term prescription**	Worries and expectations
**Knowledge about the treatment Support from friends and family members**				

### Balancing priorities: adhering to the long-term treatment

Like the findings of our meta-synthesis, the participants in this study spoke about their perception of, commitment to, and trust in the treatment; support from family, friends, and co-workers or more formal support groups; relationship with healthcare providers and professional support available to them; knowledge about the treatment; experience of side effects and of their management; adherence to medication; (non-)payment for treatment; cancer and society; and end of their treatment. A summary of these themes and corresponding quotes are provided in Appendix (Table S2). Additionally, we identified new themes or dimensions of existing themes through these interviews, enabling us to update the existing paradigm model, as outlined here.

First, in terms of causal conditions, siloed working was viewed as a problem, especially when some doctors refused to review endocrine therapy because it had been prescribed initially by an oncologist. This was related to a second factor, the view that some health professionals, including GPs, lacked the knowledge to deal effectively with breast cancer-related matters. A third factor was women’s self-professed hesitation to ask for help, for example for fear of taking up time that can be devoted to someone else. A fourth causal condition was the presence of other priorities in women’s lives including work, motherhood, and supporting others. A fifth causal condition was the bureaucracy that women encountered, and had to deal with, to progress their care. A sixth concern was uncertainty about whether women had reached the end of their treatment with endocrine medication. A final causal condition was the depiction of cancer in society, and whether discussing a cancer diagnosis was considered taboo or acceptable.

We also identified additional actions and interactions, including the monitoring of side effects, changing medication to a different brand, asking for help and support, keeping a journal, and taking the lead on arranging appointments. Additional consequences for women included a sense that their cancer took less of a priority compared to other commitments such as motherhood, work, or supporting others. A consequence of the bureaucracy women faced was that they sometimes felt lost within the system or remained unaware of important tests or milestones. Finally, some women preferred to keep their cancer diagnosis hidden from others due to a sense of cancer being a taboo. The updated paradigm model is shown in Table 3.

**Table 3:** The paradigm model for the category ‘Balancing priorities: adhering to the long-term treatment’ (verified and corrected)

Context: Accepting a prescription for adjuvant endocrine therapy *Women are prescribed tamoxifen or aromatase inhibitors as adjuvant therapy after surgery, radiation, or chemotherapy for breast cancer. Guidelines recommend the use of tamoxifen in pre- and post-menopausal women for five years and could be extended more than that if needed. Also, the extended use of aromatase inhibitors after the initial five years after diagnosis has been encouraged in post-menopausal women*				
	Causal conditions	Actions/interactions	Consequences	
Ability to adapt to the side effects of the treatment	Trust and belief in the treatment and its necessity versus fear of treatment and its side effects Wanting to continue living cancer free (realising necessity of the treatment) and fearing cancer recurrence (anticipating regret) Receiving correct information about the treatment and side effects in advance Need for knowledge vs preference for not knowing (psychological burden) Severity of side effects experienced or feared (*e.g. menopausal or psychological*) Ease of access and availability of professional support and perceived their trustworthiness Siloed working Limit of health providers’ knowledge Wanting support from family, friends, co-workers, and other patients Obligations to family to get well and owing it to others to live Ability to ask for help (fear of being burden) The perception of the treatment (positive or negative) Continue feeling as a cancer patient throughout the treatment, even though being told that they are cured, and cancer is completely gone Ability to always remember to fill the prescription and take the medication as prescribed Changes in the patient’s usual routine Expense of the medications (insurance issues) Having other priorities (work, motherhood, supporting others) Bureaucracy in the healthcare system Uncertainty about reaching end of treatment How cancer is depicted in a society	Looking for appropriate support from specialists, GPs, nurses, pharmacists, support groups, family, and friends Looking for other sources of information Trying to manage the side effects Monitoring the side effects Experimenting with alternative medicine Discussing the possibility of changing the endocrine therapy medication or changing to a different brand Asking for help and support Modifying life to adapt to the treatment and its side effects (*e.g. quitting work due to lack of energy, downsizing, changing other routines such as sport/exercise, social activities, traveling, housework, and frequency of sexual intercourse, taking up healthier eating habits)* The use of coping mechanisms to ease the experience (e.g. active coping and self-motivation, seeking physical and emotional support, maintaining a positive attitude, meditating, acceptance, humour) Incorporating medication into routine and watching for changes in usual routine Keeping a journal to record all breast cancer-related issues Asking for tests, important dates, and appointments	Adhering to the treatment despite being surprised by the challenges and the severity of the side effects (i.e. finding adherence to be more difficult than originally thought) Forgetting to take the treatment as prescribed occasionally or taking a drug holiday to manage side effects Committing to finishing the whole duration of the treatment Putting up with side effects of the treatment Suffering from the side effects of the treatment Restricting social activities Side effects of the treatment, old age, and other medications get entangled Cancer and feeling ill linger throughout the treatment Cancer treatment taking a back seat to other priorities Feeling lost dealing with the bureaucracy in the healthcare system Missing out on important tests or being unaware of important milestones Keeping cancer diagnosis a secret **Balancing priorities: adhering to the long-term treatment**	Knowledge about the treatment
**Support throughout the treatment**				

### Taking a chance: stopping the treatment early

Like the findings of our meta-synthesis, the participants in this study spoke about stopping treatment early because of the severity of the treatments and the poor quality of life while on medication, stopping the medication in the advice of others, lack of trust in their treatment, taking a chance by stopping their treatment, and benefiting from their treatment’s end. A summary of these themes and corresponding quotes are provided in the Appendix (Table S3). We identified an additional causal condition, enabling us to update the existing paradigm model. This was the idea that a completely different priority in life might be the reason for stopping the treatment, such as wanting to have a child. The updated paradigm model is shown in Table 4.

**Table 4:** The paradigm model for the category ‘Taking a chance: stopping the treatment early’

Context: Adhering to the medication and experiencing the side effects *After starting the treatment and committing to adhere, women start to experience the medication side effects, which is unexpected or more severe than they had imagined or expected*				
	Causal conditions	Actions/interactions	Consequences	
Quality of life taking precedence over longevity of life	Severity of the treatment severe side effects Poor quality of life No trust in the treatment (i.e. negative perceptions of the treatment) Fear of the possible side effects Being given the choice to stop the treatment by the healthcare provider Faith and religion A sense that existing adherence has already conferred therapeutic benefit Lack of support during the treatment Lack of trust in the healthcare providers and the medical system Having a different priority	Communication with healthcare providers and deciding to stop the treatment Stopping the treatment without communicating with anyone	Stopping the treatment early Accepting that death is not the worst option Better quality of life Regaining control Having a sense of normalcy **Taking a chance: stopping the treatment early**	Continuous search for normalcy
**Beliefs about the treatment’s necessity**				

### Core category: Hobson’s choice or a horned dilemma?

The core category we identified in our meta-synthesis was ‘Hobson’s choice or a horned dilemma?’. The findings in this study, in essence, match those identified earlier. Therefore, some participants did not think they had a choice about whether to take their treatment, the decision being akin to Hobson’s choice, given that the alternative to taking the treatment was, effectively, ‘dying early’. On the other hand, some participants struggled daily with the decision to continue or stop the treatment, akin to a horned dilemma. However, there were two participants who took the decision to stop treatment after having taken it for 5 years. Their perception of the treatment had changed from a Hobson’s choice to a horned dilemma over time, resulting in discontinuation. A summary of the corresponding quotes is provided in the Appendix (Table S4).

## Discussion

The findings of this study support our previously published grounded theory meta-synthesis. The interviews also enabled us to identify new themes or new dimensions of existing themes, including women’s ability to understand the information provided to them, the importance of support from family and friends, the impact of health providers’ siloed working and limits on knowledge, women’s ability to ask for help, balance their priorities, and tackle healthcare bureaucracy, uncertainty about reaching the end of treatment, and views on the social acceptability of their cancer. We also identified additional actions taken by women to manage their own care, such as changing their medication to a different brand, actively asking for help and appointments, and keeping a journey of their experiences. Importantly, rather than conceptualising women’s overall experiences with their adjuvant endocrine therapy as two static categories as previously within our grounded theory, the interviews also allowed us to capture the changing nature of experiences as follows. It is true that some women adhere to their treatment throughout, believing the decision to be akin to Hobson’s choice, no choice at all, and this remains the same. It is also true that others, perhaps with weaker starting beliefs about the treatment’s necessity, see the choice to take their medication as a horned dilemma throughout, especially on experiencing the side effects. The difference we found on speaking with women in this study is that there are also some who start their treatment thinking of it as Hobson’s choice, but later see it as a horned dilemma, the experience of the side effects now outweighing the perceived benefits. This change in perception is an active process which unfolds as women experience the side effects of treatment over time. This insight provides an opportunity for health providers to intervene, and potentially influence women’s waning perceptions of the necessity of their treatment.

The findings also suggest that women who are knowledgeable about their treatment or have access to someone who is well-informed might have a better overall experience. For example, knowledge about the treatment and potential side effects enabled some women to take the lead on requesting tests such as bone density scans. Knowledge is also important in relation to the severe side effects that some women experience, including knowing how to develop strategies for managing them or switching treatment instead. Research has shown that informing women with breast cancer about the mode of action and potential side effects of endocrine therapy improves their adherence, at least in the short term [11]. The support of friends and family members during the initial stage of endocrine therapy too is a factor that plays an important role in participants accepting the endocrine therapy prescription. Other forms of support include support groups, support from healthcare providers, and support from employers and co-workers, not just at the beginning but throughout the treatment. Participants’ stance about support groups varied, some finding them helpful, others preferring not to become involved in any, instead drawing on support given through their social networks. The importance of support in breast cancer is reported in multiple other studies too [12–14] while our findings are also consistent with research that also shows some women’s reluctance to engage in the formal support groups [15].

The lack of trust and belief in GPs’ knowledge and in their ability to provide the necessary help and support to breast cancer survivors within the community has been highlighted elsewhere too [16]. The literature also shows confusion on the part of GPs themselves on what type of post-cancer care they should provide [17], which has been attributed to workload [17, 18] or due to the lack of communication between GPs and prescribing specialists [17]. The women participants in our study certainly saw a reluctance in GPs to ‘interfere in someone else’s prescription’. Many women appeared also to find it difficult to speak up more generally about their cancer and considered the topic to remain somewhat taboo. The experience of unsupportive social interactions following a breast cancer diagnosis has certainly highlighted the negative impact on women’s psychological wellbeing [19]. A deeper insight has also been presented whereby women have been found to present public accounts that are driven by expectations of positivity and fear of stigmatisation throughout their breast cancer treatment and beyond [20], further highlighting the complexity of opening up about cancer.

A limitation of this study was that three of the interviews were completed via the telephone, instead of face-to-face. During these three conversations, there were more requests for clarifications, with interviewees checking the adequacy of their responses on numerous occasions. Perhaps for this reason, the telephone interviews lasted longer than the face-to-face interviews. However, anecdotally, and perhaps because of his gender as a male researcher, the first author found the telephone interviews facilitated a more open discussion of potentially sensitive topics. An informative paper published since our grounded theory study has identified a range of supportive measures in this area which include education, developing a strong personal rational for use, being prepared for side effects and having side effect management strategies, reciprocal communication between patients and health professionals, and accessible resources [21]. In this with this, and following on from this paper, as future work, we plan to create schematics from the models developed and updated through the current paper, as the basis for supporting both women and health professionals to discuss these very facts by drawing on the experiences of others elicited through research.

## Conclusion

Our grounded theory on the difficulties women face when deciding on whether or not to accept long-term endocrine therapy, and their experiences afterwards was updated through primary interviews with a cohort of women in the UK. These findings provide a more comprehensive model of women’s experiences to help the development of support programmes and educational tools to inform both women and health providers to improve care in breast cancer.

## Supplementary information

Below is the link to the electronic supplementary material.

Supplementary file1 (DOCX 99 KB)
