# Supplementary material for: Hobson’s choice or a horned dilemma: a grounded theory on adherence to adjuvant endocrine therapy verified with breast cancer survivors
Source: Support Care Cancer. 2022 Nov 7;30(12):10127–36. doi: 10.1007/s00520-022-07435-2 (PMC9715509; doi:10.1007/s00520-022-07435-2)
Supplement: Supplementary file 1 — Supplementary file1 (DOCX 99 KB) [file 520_2022_7435_MOESM1_ESM.docx]

Appendix

**Table S1. Themes, their explanations and example quotes relating to the first category: *Guided by the doctors: accepting the long-term prescription***

| New treatment stage | Example quotes |
| --- | --- |
| The women participants recognized the transition into a new stage of the treatment and talked about how that transition made them feel. Some felt “isolated” and “on your own”, and others, while recognizing the change “did not think much of it”. While some of the women spoke of feeling alone, they differed on how this made them feel. Some felt the transition was easy while others found it hard, though still managing to cope with it. | “Suddenly you’re just completely on your own, yes, and it is hard because you’re taking new medication, you’re just getting over the chemotherapy, you’ve had phone calls from your oncology team, from the chemo team then all, then that’s it you’re just left to get on with it really.” Interview 3  “You’re not necessarily thinking clearly and you’re also, and I think it’s once you pull away from the regular hospital visits and the treatment and things and reviews that you do feel a bit more isolated and on your own in terms of, I suppose, support. It didn’t inconvenience me in any way, so I suppose I found the transition quite easy.” Interview 1  “I think it could be, transitions with ease but not necessarily, it affected my life but I, but this is my face, but it’s not, it hadn’t been easy, so you can cope even though it’s bloody hard, yeah.” Interview 5  “But the transition, to be honest, I didn’t think much of it.” Interview 8 |
| The prescription visit experience |  |
| The first prescription visit (when the hormonal therapy is first prescribed) proved to be different from one woman to another. Some felt the experience was positive, while others had issues. One woman mentioned the lack of time in her prescription visit, talking about not actually being able to see her healthcare provider for long enough to go through everything she needed to relate to her treatment. | “I did ask but not many because my appointment was quite short to be honest, so I didn’t have much time with the doctor to go through everything.” Interview 8 |
| Another issue raised was that of healthcare providers expecting women to make decisions about the treatment on the spot, without having enough time to process what they are going through. | “I was expected to make a decision there and then and so it was quite intimidating really.” Interview 1  “He was saying, it was quite quick, do you have anything else? So I didn’t have enough time to process it myself and then ask, so yeah not really then.” Interview 8 |
| Due to lack of time and how overwhelming the experience could be, women reported their inability to ask questions during the prescription visit. | “Not really. No. That was it. That was the treatment and that’s what I had to go on.” Interview 6 |
| Of course, not everyone reported similar experiences. To some, the process was positive, with opportunity being given to ask and receive answers to whatever questions they had. | “Yeah, yeah it was a very, yeah I could just ask questions of the oncologist.” Interview 7  “Yes, I was able to ask questions.” Interview 9  “Yes they, I must admit the consultants were really, really good, even if it meant the clinics were late, they didn’t ever rush you, so there was time to ask questions.” Interview 12 |
| Knowledge and information |  |
| Women at this stage of the treatment receive a lot of information, both verbally from the oncologist and cancer nurses, or in the form of leaflets. Being bombarded with a lot of information about breast cancer and hormone therapy made some women in my study feel overwhelmed and confused. | “The leaflets that come with the tablets because sometimes, they can be a bit overwhelming. There’s too much information on there. This might happen, that might happen, this effect might happen, they could do this or they could do that. You need somebody that’s going to talk in, probably sounds like I’m being a bit ignorant here, but in my layman’s terms? You know, in a vocabulary that I’m going to understand without having to sit and analyse and try and make out what these instructions sheets mean.” Interview 6  “I was given a lot of, this includes all information about, I was given lots of information, booklets from Macmillan, from the hospital, everything all about the treatment.” Interview 9 |
| Another participant talked about how overwhelming she found not the amount of information *per se*, but rather the whole experience, the combination of finishing the initial treatment and transitioning into a new stage, the feeling of everything coming together at once. | “I don’t think the amount of information was necessarily too much because it’s about one lot of treatment. But when you’ve gone through operations, chemotherapy, radiotherapy and your head is full of information, maybe it’s just the tip of the iceberg and you just don’t take in quite so much. I know for me, it was just kind of, what’s next, what’s next, what’s next, you understand that these things have to happen and people take you through them but you just think, yeah that’s fine, let’s just get on with it. So I think yes, overwhelmed about the information they gave me on the tablets, probably not about that, but overwhelmed by the whole experience, so that my understanding was maybe a little bit not as good as it could have been.” Interview 12 |
| Some women really valued receiving the large amount of information, dealing with it well and feeling it helped inform their decision about whether or not to go ahead with the treatment. | “No I think the information’s been, all my care’s been brilliant.” Interview 10 |
| One participant, asked whether the information she received was too much, answered in the affirmative but nevertheless used it as a driving force to look beyond what she was given and identify further knowledge. | “Yes, yeah. And then I read, when you’re academic you’ll just go online and then just read whatever is out there.” Interview 8 |
| Another participant talked about receiving too much information and the importance of support in dealing with it, talking how having her husband with her during the prescription visit helped her to remember and deal with the amount of information she received. | “Yes. I was quite lucky, that I think, I had a lot of information, perhaps a bit too much maybe if anything, but my husband went to all the appointments with me, so there was two of us to listen.” Interview 2 |
| While some women received a lot of information, too much in some instances, others complained about not receiving enough. | “First of all they said you’d be on it for five years and, but they don’t really explain. I didn’t really know who to ask because, as I say, the oncology team are quite rushed, and you’ve gone through your treatment, so you just feel as if they’re giving you them.” Interview 3  “I wished it had been explained a little bit more, with the side-effects of them. What to expect and also, whether there was any other options of drugs that I could have taken.” Interview 6 |
| Others preferred to be “spoon fed” information gradually, fearing they would be overwhelmed if given it all at once. | “They didn’t tell me all of that to begin with and afterwards I thought, I felt as though they should, but I think they do that so that you’re not too overloaded with everything and they just, sort of slows it, well you're going to have to take this medication for ten years. Maybe if they told you all that at the beginning it would be too much to cope with. I was a bit spoon fed, which I think was probably the best thing really.” Interview 10 |
| While healthcare providers often try to give patients as much information as they can, without focusing on the patients’ personal preference, identifying each patient’s needs and health literacy level is indispensable in delivering a good service. One participant spoke of the need for a tailored way of delivering information in ways suited to each woman’s particular needs. | “So it’s too much information in one way and not enough in another I think, it’s an imbalance. It’s not tailored at all. And a lot of it’s very medicalized, the information that you get. And that’s alright for me, but it’s not alright for a lot of people.” Interview 14 |
| When participants were asked about their primary source of information, and who they would contact if they needed questions answered in order to make informed decisions, their responses varied. While some contacted their healthcare provider, others went online to search for answers. “Doctor Google”, a term used by one participant, is mentioned multiple times in the data. Another participant talked about identifying academic papers, still another about breast cancer and other cancer-specific websites as their primary source of information. | “There’s doctor Google of course − it’s huge isn’t it?” Interview 3  “I’ll look at Google Scholar, and look at academic ones, so I will try and go into medical journals, actually, because I can kind of understand most of what they say, although I don’t understand the medical language. But the introduction, the conclusion, you can get a sense of that.” Interview 4  “I went to breast cancer, there is a website breast cancer and then there is another one, the Macmillan one.” Interview 8 |
| Support groups were mentioned as a source of information by some women. After the diagnosis and the consultation, some women contacted their local breast cancer support group in an attempt to learn more about the treatment and their options, especially if their healthcare provider had left them confused or uncertain. | “When they’re diagnosed the surgeon or the oncologist gives them as much information as they can, obviously, but unfortunately, a) you don’t always take it in and b) they don’t give you a lot of information about taking Arimidex or tamoxifen, simply to take it and it will help with trying to prevent it. So that’s about as much as you get these days and they just, they don’t even say, make sure you read the leaflets, they, which would just take a moment or two. I recognize timing is always a problem with the consultants but it would be a good idea just to emphasize the fact they should read them. Most people would recognize that but you’re not thinking very straight when you’ve had a diagnosis of breast cancer.” Interview 13 |
| Delaying the start of the treatment |  |
| When asked about whether or not they thought about delaying the treatment, most participants said no, some making clear their wish to start it as soon as possible. | “It wasn’t something I considered, delaying treatment. I just wanted to get through the treatment and get back to work.” Interview 1  “I was very keen to get on with it.” interview 2 |
| One participant mentioned taking a break for two or three days before starting the hormonal therapy treatment, after getting her healthcare provider’s approval. To her, the priority was going back to her normal, medication-free life for a couple of days before embarking on the journey of hormone therapy. | “I said OK I’ll give myself two, three days to say, I’m free of treatment at the moment and then I’ll start with it, yeah.” Interview 8 |
| Another woman, with the blessing of her GP, decided to wait until she had finished the chemotherapy before starting hormonal treatment. | “I didn’t start it when I was going to start it because my GP did say let’s hold off for a while because you’re still having your chemo. You don’t want that as well.” Interview 10 |
| Necessity of the treatment |  |
| Some women believed the treatment to be necessary; to them, taking the treatment was a small price to pay, taking into account its associated benefits. | “So I just reckoned, again doing a cost benefit analysis, that it was worth taking the medication.” Interview 4 |
| Women start the treatment because they understand its importance and the fact that it decreases the chance of cancer recurrence. When asked about their main reason for taking the treatment, most replied on the lines of understanding the importance of the treatment and the fact that it was their best option of preventing cancer returning. | “Because they said it was the best chance I had of it not coming back.” Interview 6  “Well the main reason that I decided to go through that is because they said that you reduce the risk of cancer coming back, so that was the main motivation to be honest to do it.” Interview 8  “I just thought, well yeah, I’ll take it, because obviously I don’t want to get the cancer back again.” Interview 10 |
| Fear and worries |  |
| Difficulties during the initial stage of the treatment affected how the participants perceived the medication. A participant was afraid of the nausea before starting hormonal therapy due to the fact that she suffered from it during the initial stage of the treatment. The same quote shows the effect of others’ experience on the participant’s perception of the treatment; she is talking about her friend’s bad experience with the side-effects of hormonal therapy and how this made her anxious about starting her own medication. | “Yes, I had one friend who had struggled with it because I had two friends I knew who were taking it and one had terrible nausea. And my problems, I had very, very bad nausea during chemo and also, I had and in fact, ended up with sepsis during chemo as well and various other complications. So, I was anxious about taking it.” Interview 9  “I wasn’t fearful of doing it but I, in a very weak state because the chemotherapy affected me very, very badly; surgery and radiotherapy were a walk in the park.” Interview 14 |
| Fear of recurrence is evident from many of the interviews. Having gone through the initial stage of the treatment, the women did not want to relive the same experience, deciding to take the treatment to make sure they would not have to go through it. When asked about the main reason for taking the treatment, one woman answered it was fear of cancer. | “Fear of cancer.” Interview 9 |
| Fear of death is also noticeable in many interviews. This participant considered the treatment her best option of staying alive; to her, the necessity of the treatment was associated above all with its ability to keep her alive and well for as long as possible. | “I didn’t want to die. I didn’t want to die just yet. And, yeah, I just didn’t want to die just yet. I’ve got too much to do.” Interview 11 |
| Fear of the possible side-effects of the treatment is also to be observed in the data. Despite going ahead with the treatment, women still feared its long-term effects and what it might do to their bodies. | “And then they gave me all the instructions and what are the side-effects of this medicine and so on. So when I read all this I said, oh no what is coming?” Interview 8  “It’s six years’ ago. I think that I was anxious about how I would feel taking it. I was anxious about the side-effects.” Interview 9 |
| As suggested, this fear did not necessarily lead to a decision not to take the treatment; for many, side-effects were a small price to pay for the benefits the treatment could provide. | “No, not really because I suppose I felt, well, I was told that there would be side-effects with the anastrozole, or possible side-effects, but for me, if the worries about a few side-effects and prolonging my life was, it’s, it, that was, that was the benefit.” Interview 11 |
| One worry some women had at the prescription stage had to do with whether they were being prescribed the correct treatment. Another concern of some was a lack of certainty about whether or not they were menopausal, making them wonder whether they should be prescribed tamoxifen or an aromatase inhibitor. | “I did have some concerns because it was a drug that had to be taken, you already have had to have gone through the menopause to have taken that as opposed to Tamoxifen and I was, well have I, haven’t I, how do, you know, how do I, I was just borderline on that. Am I taking the right drug, so yeah, I did have some concerns yes.” Interview 1  “I don’t know, because I, they told me no this isn’t right, but I wasn’t menopausal when I was diagnosed with the cancer and also I wasn’t fully menopausal after chemotherapy, radiotherapy, taking Tamoxifen, so I obviously wasn’t ready to have the menopause.” Interview 5  “I'd previously been told I would have letrozole, they said oh, we, I don’t think we can do that, we’d better give you tamoxifen and I knew from looking at the literature that letrozole was a bit better.” Interview 14 |
| Vulnerability |  |
| An important aspect to cover at this stage is vulnerability. To some, becoming a patient and having to go through so many procedures and medications was a novel experience that made them feel vulnerable. | “I did feel very vulnerable because I’d always been a very well person and I think people who haven’t experienced a serious illness are perhaps not aware of how vulnerable you feel when you are diagnosed with something.” Interview 2  “I felt vulnerable having not been a patient before and having all these procedures.” Interview 7 |
| Another thing that made some women feel vulnerable was having to go through body changes while they were in the public eye. To some, enduring cancer was not a topic they wished to be visible to others. | “Vulnerability I think is key, and I would say as an academic, somebody who appears in public, who considers herself to be a very capable person, going through that vulnerability is something, is one of the biggest life changes you have to deal with.” Interview 4 |

**Table S1. Themes, their explanations and example quotes relating to the second category:** ***Balancing priorities: adhering to the long-term treatment***

| Commitment | Example quotes |
| --- | --- |
| Generally, women in the study tried to adhere to the treatment as best they could. They were committed to the treatment plan. | “I just thought, well no, I know it’s helping me so I’m going to just keep going.” Interview 5  “Once somebody advises me that you have to go through that then I say, yeah OK, this is what I need to do so yeah.” Interview 8  “I think psychologically, it probably wouldn't have made any difference if I'd stopped a few months earlier, but psychologically I had set myself that goal, it was like getting to the top of Everest.” Interview 14 |
| A factor that plays an important role in participant commitment is the fear of recurrence. The data show a clear fear of cancer coming back, with some participants recognizing it as the driving force behind their decision in favour of taking the treatment. Participants wanted to continue living cancer free, leading them to look upon the treatment and its side-effects as a small price to pay for what the medication could potentially provide. | “At that time you just don’t want it to come back so you just think, well if I don’t have the hormone for the cancer to feed on it’s, the likelihood is it won’t come back and I think that’s the more, the forefront of your mind you just don’t want the cancer to return so you take anything.” Interview 3  “Well, the main reason would be to prevent the hormone, because my cancer was hormone receptive, it’s to prevent the cancer coming back.” Interview 4  “Because at the time − I don’t know if it’s changed now − but at the time there was no chemotherapy for my type of breast cancer. I was more worried that if it came back then maybe I’d be stuffed, for want of a better word. So I would rather put up with any minor side-effects and reduce that risk, so…” Interview 7  “I still have this sense that anything to stop it coming back is worth it. And there are other side-effects that I also don’t like but in comparison to a recurrence, you put up with it.” Interview 9 |
| Some women showed commitment to take the treatment as prescribed for their family’s sake, feeling as if they owed it to them to adhere and to do all they could to prevent recurrence. | “I have two kids, so yeah, initially when I was in the previous stage, considering whether I go through this treatment or not, then I will say, yeah, I had to do it for my kids, so yeah that happens.” Interview 8  “Because it’s not just for yourself that you’re taking these tablets, it’s for your family.” Interview 13 |
| Although in some cases the side-effects were very severe, some women, showing great commitment, continued taking the treatment. One participant quoted here judged the trade-off to be well worth it and regarded suffering a few side-effects as nothing by comparison with what the treatment offers. Another participant who was suffering from very severe side-effects talked about experiencing them as confirmation that the treatment was working and doing what it was supposed to do. | “So, yeah, the volcanic moments and the night sweats were a bit, a bit of a bugger, but better to put up with something that’s a bit inconvenient and be alive than not, was my view.” Interview 11  “I did read some encouraging research that the more severe the symptoms the better the prognosis. I don’t know how true that is, but I thought, oh, it’s working.” Interview 14 |
| Trust in the treatment |  |
| Participants generally trusted that the treatment was their best option to prevent cancer recurrence and showed readiness to bear the side-effects no matter how severe they might get, due to their belief in the treatment’s necessity. | “I saw it as a positive thing because I, you knew they found a treatment that, I wouldn’t have had to take it unless it was going to be beneficial and I was fortunate that I was hormone receptor positive to that sort of treatment.” Interview 1  “It was necessary to prolong my life. Otherwise if I hadn’t had the treatment, I had a life-threatening illness.” Interview 2  “I have but it comes back to the fact it could prolong my life and the side-effects, hopefully, when I come off of it, will go.” Interview 6 |
| Perception of the treatment |  |
| Perception of undergoing the hormone therapy varied from one participant to the next. While some looked on it as an easier experience by comparison with what they had endured during the acute stage of the treatment, others took a different view, considering it an even less pleasant experience. | “Yeah, so I think the surgery was really the big thing and also knowing that I’d need chemotherapy as well and then taking the Tamoxifen was a third thing, but it was of less, lesser significance to me at the time because I found the other two pieces of news quite upsetting.” Interview 2  “Yeah, yeah, it is really, because, all right the chemotherapy you lose your hair, but I was lucky, I didn’t feel sick, I didn’t have any side-effects other than obviously you get very tired towards the end, but for a lot of it I felt fine, yeah. Although you do start to have hot flushes with the chemotherapy because obviously it’s knocking on the hormones isn’t it, but not as much as with the Tamoxifen, because you can imagine having a hot flush every 20 minutes and they last quite a long time and it goes on for 24 hours a day, so it’s not like it stops at night. It disturbs your sleep so for, since I’ve been on that, I always have disturbed sleep. I don’t sleep soundly because you’re having the hot flushes all the time, yeah. They have worn off but I, not completely, but assume that’s to do with my age, so yeah.” Interview 5 |
| Participants’ perception of the treatment was shown to be strongly side-effects-related. When asked if their perception had changed over the time of taking the treatment, their responses showed side-effects to be an influential factor, whether positive or negative: | “Well, my perception slightly changed simply because I didn’t see all the side-effects happen and I said, OK fine it’s just an extra pill, so…” Interview 8  “Well yes, because I didn’t know that it was going to be, have the side-effects. They don't tell you the side-effects then, they tell you you’ve got to take it, they give you the leaflet but and my GP just prescribed it, she didn't talk to me about it.” Interview 10 |
| Some participants talked about the fact that they still felt themselves to be a ‘patient’ while taking hormone therapy. Despite being cancer free, taking the hormone therapy pill every day served as a constant reminder of what they had to endure. | “I do feel I’m in remission and the longer it goes on the better really, but I, once you’ve been a cancer sufferer, you’re always a cancer sufferer in your mind.” Interview 2  “From, well obviously I'm only six months on this thing, but still I feel like, even if I had, I had my surgery and all this stuff, I had radiotherapy, I started hormone treatment but I still felt sick. So yeah.” Interview 8  “Well yeah when I take it every morning it does remind me that I've had breast cancer, yeah. So yeah it does, it does remind you.” Interview 10 |
| Another participant thought the treatment and taking a pill every day was not a big deal, feeling that because she was already taking medications daily, one more pill was not a problem. | “So every morning I’m taking Thyroxine so it was just an extra pill so I took 75mg so I had one more. So it didn’t change much of my routine to be honest, instead of two I was taking three so I said, fine. So that’s why I didn’t find it hard to start taking a pill every day.” Interview 8 |
| Support from family, friends and co-workers |  |
| Support from family and friends was felt to be important during the treatment, helping participants cope better with the experience. | “My friends are very good and very supportive. I have a very close friend who works in the Intensive Care and she has been my rock really.” Interview 6  “I mentioned family but my friends were just completely amazing.” Interview 10  “I just don’t tell him often enough, they, they’ve been there when I've wanted them to be and friends have done that as well. They’ve, friends from a long time ago have come back into my life because they thought I needed them.” Interview 12 |
| Other participants mentioned the inability of some friends and family to offer help and support. Reasons referred to included people being busy with their own lives and issues, and in other cases an inability to deal with the cancer diagnosis. | “Some of them couldn’t cope with it as well as others, because some people have their own, one of my quite close friends she couldn’t cope with it because her daughter had gone through leukaemia when she was about 12 and she couldn’t, she couldn’t cope with supporting me because it just brought it all back, which I completely understood.” Interview 10  “Some friends I've lost completely because they couldn’t cope with it.” Interview 12  “They couldn't do this and they couldn't come and live with me to look after, I didn’t needed looking after I needed nursing but they couldn’t give up their lives, their work, their, looking after their families and people who are ill in their own families.” Interview 14 |
| Some participants mentioned how helpful they found it to have a friend who had survived breast cancer and gone through a similar journey to their own. To them, having someone who understood what they were going through, and with whom they were able to talk to freely and openly, felt “great”. Having someone close to them who was able to offer advice from their own experience gave these participants a feeling of security, making them better able to cope with the treatment and its side-effects. | “When I was going through cancer, a friend of mine, who’d also been through it, was, her advice and help was enormously helpful to me. So I would say that the support of other people, who’ve been through what you’re going through, is enormously useful.” Interview 4  “I was so lucky when I went through my breast cancer because I had a friend who is sadly going through, who had already gone through and sadly she had mets so she didn’t make it but I was so fortunate to have that, someone to hold my hand and talk back, quite openly talk to her about whatever I felt and I just, I know how that made me feel. It made me feel so much more secure and able to cope that I just felt that you want to give back really.” Interview 13 |
| One participant talked about the importance of empathy. For her, despite her family and friends not being able to provide her with the physical support she needed, the empathy and understanding they offered was enough to make her feel better. Another participant talked about how some people displayed a lack of empathy toward taking hormone therapy. She felt that the issues women experience while on hormone therapy are poorly known, with the fact that they (the participants) appear normal on the outside, making some people dismiss what they are going through. | “That was the support I, I had the empathy of my friends and family.” Interview 14  “I don’t think the hormone therapy is very publicized, people laugh at hot flushes, but they can be really, my sleep pattern at night is dreadful − it’s off, on, off, on and you’re tired, I feel tired. I think when you physically look different people feel sorry for you when you’ve got no hair and your face is all bloated because of the steroids and they’re, oh, but then when you start looking better I think people soon forget.” Interview 3 |
| When asked about the support received from family members, one participant talked about the fact that offering support was *her* role as a mother. Similarly, another participant mentioned that she did not feel comfortable taking about cancer with her family, because protection and support are “part of being a mum and a wife”. To these participants, motherhood took precedence over breast cancer and their need for support. | “Yeah, and also my, I had, my daughter had a very serious problem with her eyes and I think when you’ve had children, you really worry about them, and when, and I had many sleepless nights pacing the floor with adrenalin with her, when I had my cancer diagnosis I didn’t even lose a night’s sleep with it, so I think it’s having kids. Also my children would have been able to look, they were older, they weren’t babies, so I think that made me, and also it’s just my nature. I’m just somebody that’s going to roll with it if you like, so yeah.” Interview 5  “But sometimes it’s not easy to talk to family because you, naturally, you want to protect them. So, that’s what you do. That’s part of being a mum and a wife isn’t it? So, yeah.” Interview 6 |
| Fear of being a burden on their close ones was another factor reported by one participant as the reason she felt unable to ask her family and friends for help. She preferred to deal with cancer on her own rather than trouble someone else with her issues. | “Yeah, I am and I have this concept that, it’s my problem so I have to deal with it. I shouldn’t burden other people. All through my cancer, the only way I could deal with it was, every time they told me something, we used to say, put it in the jam jar and screw on the lid, and that’s where it stays. Because, I knew my family couldn’t cope with it and it’s not fair to burden other people, so, yeah, no, I don’t look for support.” Interview 6 |
| Support at work was an important factor reported as easing some participants’ experience on the treatment, although here again support levels varied. While one participant here recalls about how supportive and understanding her employers were, another talks about the fact that some women might find it difficult to discuss their diagnosis with their employer for fear of losing their job. | “Also, I would have to say that my, my work colleagues were supportive and HR worked very carefully with me. So I was able, during that time, to do flexible working and that was enormously helpful.” Interview 4  “Yeah and some people worry about losing their job if they tell their employers.” Interview 14 |
| Support groups |  |
| The experience with support groups was different from one participant to another. Some women liked being involved with support groups while others did not. One participant talked about how helpful it felt to share things with women who were going through a similar experience. Another mentioned meeting another breast cancer survivor at the support group and then becoming very close friends, helping each other throughout the treatment. | “Yeah well it does help to know that other people feel like they're 90 when they get up as well, not just me. [Laughter.] I think we all discuss how horrible it is.” Interview 10  “I am in touch with the breast support group that she runs and I went to it before I started chemotherapy and met somebody that I kept in contact with that I found, she’d been through things and so we’re quite friendly now, we don’t see each other quite so often but we keep in touch.” Interview 12 |
| Others, when asked about support groups, were quite reluctant to speak. One participant said that attending such groups and sharing her experiences with others was simply not in her makeup. Another went to a support group once but felt out of place as all the other women there knew each other. A third participant went to a support group but found going to the meetings difficult and that even when she went, they were not able to offer the help she needed due to the severity of her side-effects. | “To be honest I’m not, it’s not in my character to go in these groups and start sharing my experience so far. I don’t like it anyway so that’s why I didn’t try to get involved a bit further.” Interview 8  “I went to one and I thought that would be good, but they all knew each other very well and they were all of, they were all a bit older than me and they were of a different age. And they weren’t, they were very nice people but they weren’t professional people.” Interview 9  “I did eventually join a Breast Care Support group, but they don’t really, well if, be well enough to get there for a start, but there were no support groups specifically for this.” Interview 14 |
| One participant shared that she did not know about the availability of support groups and thought that information should have been provided to her when she was prescribed the medication. | “No, I haven’t. I wouldn’t even know where to go to actually look for one. That may be something that would be good to be introduced in the early stages of when you are actually prescribed this medication.” Interview 6 |
| One participant who worked at a support group shared her experience in the interview, speaking about the help she was able to provide by answering survivors’ worries and putting their minds at ease and how fulfilling she found the experience of providing others with help, support and guidance. | “I’m on a helpline so sometimes I do get ladies ringing me and suggesting that the, asking about the side-effects and was it normal, etc., because you tend to, sometimes when people have side-effects they’re not necessarily clarified. Well, they’re not mentioned actually when you’re prescribed and sometimes people don’t read the leaflet they get inside and even if they do they sometimes think that the symptoms that they’re getting have got something to do with the cancer so get quite nervous about it. When someone does ring and they do have a problem and at the end of the phone call they say, oh, thank you so much, I feel so much better now. And I think to myself, well, actually I haven’t really done much at all but to hear that it’s so nice to know that you have been able to relieve their stress a little bit because it’s a big problem, the stress levels, yeah.” Interview 13 |
| Relationship with healthcare providers |  |
| Having a good relationship with healthcare providers was reported by some participants as a factor that improved their experience. One woman talked about how meeting the same healthcare provider at every visit helped her greatly. She felt that her healthcare provider was able to notice any changes, whether positive or negative, and address them accordingly. Another participant talked about establishing rapport and how being comfortable enough to share small talk with her healthcare provider helped her greatly. | “It did throughout the treatment, because she noticed any difference in me, so if she thought I looked worse or looked better she was able to see that, so it was, it was quite good that she was at my last appointment as well. I know it doesn’t, it doesn’t always happen because when I went back after a year she wasn’t there, she’d left, but certainly for me it worked really well that it was the same person.” Interview 12  “I used to like going in and talking to him. He liked motorbikes. I liked motorbikes. So we used to spend, he’d go, are you OK, and I’d go, yeah I think I’m fine, he went, right OK let me just check. He’d check and then go, and then we’d spend 20 minutes talking about motorbikes. I had a very good relationship with him. You know, in that respect, I loved him. And I also loved him because, as far as I was concerned, he saved my life.” Interview 11 |
| Other women reported a different kind of experience. One participant met with a different nurse every time she went for a visit and felt that it was almost impossible to establish rapport and build a relationship. Another participant when asked about her relationship with her healthcare provider replied “non-existent”, feeling that healthcare providers thought of her as just a number. | “Yeah, I think it is because you don’t, they say when you first get diagnosed that you’ve got your healthcare nurse, the breast cancer nurse but you never see them, it’s always someone else that you see so then you don’t really build a rapport up with anybody, that was my experience.” Interview 3  “No, yeah, non-existent. It, I felt a bit like a machine. OK, and next number please. Yeah, I don’t, I don’t think there was a relationship.” Interview 6 |
| Women reported the importance of trusting their healthcare providers. One woman talked about how, having faith in her healthcare providers helped her try and adhere to their advice as best as she could. One participant talked about not actually having a choice in the matter: she felt obliged to trust them because, as she put it, her life was in their hands. | “I had total faith in the guys that were treating me, so if they said to me, that’s what you need, that’s what I was going to do, yeah.” Interview 5  “I trusted my two consultants all the time, because I thought if you don't trust them then it’s just important to know that, feel as though they're doing the right thing.” Interview 10  “I think you have no choice. If someone’s got your life effectively in their hands, you have to trust them.” Interview 11 |
| One participant talked about losing trust in her GP, feeling that they were dismissive of her experience and lacked the understanding and knowledge to deal with her situation. | “I certainly lost trust in my GP as a consequence of how, they basically, you, they basically at a time I suppose you’re under hospital care but when you’re, there’s ongoing that you’re under the GP care as well. I felt really that I wasn’t supported by the GP, particularly when I had concerns and then they dismissed them and then I had to go back to the hospital then to find out I could then have a bone density scan, so I lost confidence in my GP throughout, because they had little contact with me.” Interview 1 |
| Some participants shared their worries about healthcare providers’ overprescribing. One talked about how her healthcare provider had been ready to prescribe her a medication she was already taking and feared that had she not notified him she might have ended up being overdosed. Another woman shared her worries that healthcare providers are linked to drug companies and that their prescription patterns are related to that fact relationship − a thought she called “cynical” but nevertheless couldn’t shake. | “I went to the doctor for something and he was going to prescribe some ibuprofen type tablet and I said, do you know that I’m meant to be taking ibuprofen anyway? He said, ‘Oh no I didn’t know that, oh no that won’t be any good for you then.’ So, you just think, actually they don’t even read your notes, they don’t even know what you’re taking because they would have overdosed me on that, wouldn’t they?” Interview 3  “One of the things I worry about is whether oncologists, I don’t know this, but whether the oncologists are linked in with certain drug companies. So that’s something I wonder about and whether, in some cases, they’re recommending treatment that doesn’t always have to be prescribed.” Interview 4 |
| Professional Support |  |
| One of the difficulties reported by participants at this stage is the bureaucracy in the healthcare system in the UK and the different hoops they had to go through to ask for tests, medications or to just meet a specialist. The ability to deal with this complicated process again differed from one participant to another and played a role in the service received. One participant talked about her complicated experience of working around the bureaucratic complications to ask for a bone density scan and the many steps and hoops she had to jump through to finally be able to do it. Another participant shared her experience of feeling lost when she had to deal with a blood clot and how, when her GP told her to go to the hospital for the prescription, she felt lost in this bureaucratic labyrinth and ended up thinking, “just give me the medication”. Another participant shared her preference for the [another country – removed to maintain anonymity] medical system with which she was familiar. There, she said, she could more easily find the healthcare provider she needed, rather than having to go through the complicated UK bureaucratic process of booking an appointment with a GP in the hope of being referred to a specialist. | “I spoke to my GP about it and having a bone density scan, and they told me that I wasn’t entitled to have one. I was concerned for two reasons. One that, well I’d had a few joint pains and aches and pains but then I thought that was, that could have been anything at my age whatever, but also because I’d had radiotherapy and I knew it was, well the risk factors with taking the treatment and it wasn’t, so I, she went through various criteria, told me I wasn’t entitled, and I went back and spoke to them in the hospital. My consultant wrote a letter and then I had a letter from the GP saying yes they’d arrange for me to have a bone density scan, and then I found that I’d osteopenia and so they prescribed alendronic acid for that.” Interview 1  “I had a blood clot in my arm because your veins started to collapse and I had to have this injections for so many months after, it was a bit like, to thin the blood and then they kept saying, oh well really the hospital should be prescribing these because of the budget, and you just think, well I don’t really mind just give me the medication. So, I think there’s a little bit, the oncology, the doctors.” Interview 3  “Yeah it would be something that I would really love to have when I have an issue, can I see the oncologist, obviously the NHS doesn't work like this. You have to go through several steps in order to reach the specialized doctor. In “their country” as well it's like this. You go to the doctor that you want, whenever you want, whenever you feel that you want to see it rather than waiting to get an appointment whenever, yeah. OK.” Interview 8 |
| This brings the issue of cooperation and communication between the different health sectors into question. Some participants shared their experience with their GP and the fact that they showed no interest in interfering with a hormone therapy prescription. Even in the case of side-effect-related issues, GPs were reluctant to offer advice and guidance and suggest that the woman should contact the oncology team that had prescribed the medication. | “It’s hard to get an appointment at the doctors anyway and as I say, I don’t really feel as if they’re that interested in this. They just say, oh yeah well the oncology prescribe them so, no one’s ever reviewed it.” Interview 3  “I tried to talk about it to my, one of the doctors at the practice, the one who takes my blood because he’s the best one, but he wasn’t, he just thought I should talk to the oncologist about it.” Interview 9 |
| Healthcare providers’ limited knowledge of the treatment was mentioned by some participants. One talked about GPs’ lack of knowledge and training in dealing with a breast cancer diagnosis, mentioning the need to provide them with the education that would enable them to it handle it better. Another woman talked about her side-effects and the fact that her healthcare providers were unable to provide her with the help she needed. She thought that they simply did not know how to handle her condition. | “Nowadays you get a 12-month follow-up following your treatment. So it means that the GP is the person that is dealing with the hormone treatment and unfortunately they really don’t have the information that they need to deal with. So again it’s about education, it’s about GP education. I mean if you talk to a GP, I do go to various conferences and lectures and what have you, and if you talk to the GPs they really do feel that you’re having a go at them all the time. And that’s not what we’re saying. We’re just saying that the powers that be need to make sure that GPs, I don't know how you do it because they do have access to the information, but they just don’t do it.” Interview 13  “I don’t think they, I don't think it crossed their minds, I don’t think they were withholding information from me, I don't think they, I don’t think they knew it.” Interview 14 |
| The same participant raised the question of the necessity of doing more research, especially since from her own experience, healthcare providers’ knowledge about the side-effects and how to manage them was clearly lacking. | “Why isn't there more research into the side-effects when they're crippling?” Interview 14 |
| Healthcare providers tend to be busy. To some participants, finding an appointment proved rather difficult, and the issue came up multiple times in the interviews. | “It’s hard to get an appointment at the doctors anyway.” Interview 3  “I don’t really routinely because they are just so busy, aren’t they?” Interview 7 |
| One of the complaints about healthcare providers was their occasional lack of compassion. Two participants mentioned how healthcare providers sometimes showed little or no compassion, and how condescending their actions could sometimes be. One of them highlighted how such behaviour could prove positively dangerous. | “They're so used to giving out the medication, they don't really think of it, of the side-effects of it, I don’t, I just don’t think they realize how horrible it is to take.” Interview 10  “A lot of GPs do not know, if you go in with a lump, even going with a lump if you’re a young woman they will suggest it’s to do with your cycle, your menstrual cycle, and will not take it seriously, OK? And women know their body. He’ll say come back next month. See how you go with it in a month and come back. Well, if you’ve got a young woman who’s got an aggressive breast cancer, that’s actually too late and I have, I’ve had ladies who have joined our support group who have died as a result of the doctors not taking their fears seriously and not referring them.” Interview 13 |
| Another participant talked about a friend of hers who was going through a similar journey and the fact that, when her friend contacted healthcare providers to ask for information, she was really looking for hope and encouragement rather than knowledge. | “She’s contacted Harley Street, because her way of dealing with it is she just wants more and more information. Basically she wants to be told if it’s going to come back or not and nobody can tell her that.” Interview 10 |
| While compassion is important, one participant talked about how a sympathetic healthcare provider was actually less helpful to her. From her own experience, she found practical healthcare providers were the ones able to provide her with better care. | “Whereas I think those who were sometimes overly sympathetic or something, it actually didn’t really help.” Interview 9 |
| One participant talked about the fact that she was assigned a breast cancer nurse to help her throughout the treatment and answer any worries she might have; however, she was not informed of her availability or how to contact her. Another woman shared her experience of not knowing that cancer nurses could have offered her help with hormone therapy-related issues. | “Well, the one thing that wasn’t helpful, that didn’t help me, was you were assigned a breast care nurse and I didn’t really make use of it because I thought I don’t have one. So, I think that was the one thing that it hadn’t been clear to me that I could contact her.” Interview 9  “I haven’t to be honest, because you do go back to your cancer nurse once a year for the first five years, so my last one was last year and then they do say that if you have any problems to come back and to contact them, but I think that’s more if you find any lumps, that’s what I assumed − I didn’t think it was related to the hormone therapy.” Interview 3 |
| Not having enough hospital visits or access to healthcare providers was reported by some participants as a factor hindering their experience. One talked about how having too few hospital visits made her question her treatment. Another shared her experience of how vulnerable it made her feel. | “I suppose with less hospital visits and less contact with professionals you begin to question things a bit more, including any medication you’re on.” Interview 1  “Yes, that was I think because I didn’t have a care worker, in a sense, or didn’t make use of, I hadn’t quite realized that I could, I think therefore I, there were fewer hospital visits, so it was yeah quite interesting, big difference. I think I possibly felt more vulnerable, probably.” Interview 9 |
| Treatment follow-up is usually on an annual basis. Participants talked about how 12 months is a long time without seeing an oncologist. Another participant talked about the fact that even during the follow-up visit, medication was not discussed at all. | “Whilst I was on the treatment I was having, obviously I was having annual check-ups and mammograms, so if I had any concerns I could have spoken about it then. It was more a case of more of a physical examination and I was asked if I had any concerns, but they didn’t specifically mention my medication. It was actually just, it was almost forgotten about, really.” Interview 1  “I don’t think so, no. I don’t think it’s changed at all. I think I thought I would have more one-to-one consultations with oncologists in the beginning, rather than six months gap, and then a 12 months gap. Where 12 months is a long time.” Interview 6 |
| Another issue regarding follow-up visits is their duration. Participants talked about how short these sessions were and the fact that, as a result, they did not have enough time to discuss their issues with their healthcare providers. | “I think the problem in this country as well now is that GPs are so stretched that they don’t have time to sit down with people to discuss things like this, you know.” Interview 7  “And so that would have helped, but I, mostly in general practice there isn't the time for that kind of discussion, usually.” Interview 14 |
| Some participants talked about the importance of keeping a journal to record all their breast cancer-related issues. Writing everything down helped them remember their experience and proved helpful when they wanted to share them with their healthcare providers during their follow-up visits. | “Because this is my little book you see, so all the way right from the beginning I wrote everything down, because I thought I can't, you can't remember.” Interview 10  “Yeah and I think that’s what I would say to people as well, is to write everything down, how you're feeling, keep a diary so that when the doctor says to you, ‘And how are you?’, you don’t say I'm fine, you can say ‘actually I'm OK now but two weeks ago I was feeling really sick’.” Interview 12 |
| Participants expressed their need for more professional support during the treatment. They talked about their need for someone to talk to who could answer their worries and help them navigate their experience. Sometimes the need for professional support is not just physical or related to issues with the treatment, as emerged when one participant talked about her need for support to ease her state of mind. | “Yeah, I think so, even if it’s just a nurse. It doesn’t have to be the doctor, it’s just someone to say, ‘How are you getting on? What are your side-effects like?’.” Interview 3  “I don’t think it’s anybody’s fault, the doctors and the oncologists, they have to see so many people that there probably isn’t the time, and, I guess, maybe my case wasn’t as severe as many others, so, though other people needed their time more than I did, but, mentally, I think I needed the time. Maybe not physically, but mentally.” Interview 6  “So, well, the support I needed was something to take the edge off the worst symptoms, to make them manageable and that really is from people like yourself and medicine and specialist nurses I think, really.” Interview 14 |
| One participant reported her inability to ask for help, feeling that picking up the phone and contacting someone to ask for information and guidance was not part of her nature. She also spoke about her fear of becoming a burden on the healthcare system was she to do so, fearing that by contacting her healthcare providers she might be taking time from someone whose need might be greater. | “Some people would find it very easy to just ring up their breast cancer nurses and say, ‘Oh, this is happening, that’s happening, what do I do, is this correct, is that correct?’ But there are other people, that that is extremely difficult, and one of those people is me. And then you feel like, you’re taking the appointment of somebody else that may be desperately need to see the doctor, whereas I’m just asking information. I feel like I’m taking their time up when there’s probably someone else that needs them more than I do.” Interview 6 |
| When asked about future visits, participants shared their experience of how these were usually booked in advance. One participant talked about how her hospital called her to arrange the follow-up visit. Another had a different experience as she usually had her appointments booked and confirmed before leaving the hospital at her previous appointment. | “Yes, they will contact me, yeah, yeah. Because my timetable is so busy I usually call them months in advance to set up my meetings. But if I don’t do that, they will call me.” Interview 4  “That I always had the next letter for the next appointment. I was never at home worrying about not being seen and that makes absolutely so much difference.” Interview 5 |
| The pharmacist’s role in the healthcare system was obscure to most participants, their experience being that pharmacists usually just deal with minor illnesses and dispense prescriptions. However, one participant talked about how her pharmacist offered her advice when she went to them with a side-effect of hormone therapy, and how that advice helped her. | “Yeah and they see pharmacists more as a, you know, to discuss minor ailments with really, rather than maybe their prescription medication.” Interview 7  “What one of the pharmacists told me, this was in the, not in the hospital but in the, because I didn’t meet, well apart from when I went to collect the initial drug I didn't meet, well it wouldn't have even been the pharmacist then I saw. One of them I asked about the headaches and he said that although a lot of people will say, with something like ibuprofen it doesn’t matter which brand you take, actually it does, because of the excipients in there and that, and then I started looking up, I did look up that information as well, that although you're always taught, no, no all these other things they're just packaging, they don't affect the patient − oh yes they can, and actually they can interfere with the action of some, I think is it particularly cardiovascular? Sometimes they can interfere with, so I started looking up all of that. And he said no, take; he recommended for the ibuprofen, the Nurofen, rather than the cheaper brands, and actually I did find a difference between the brands.” Interview 14 |
| Knowledge |  |
| Knowledge and information about the medication and its side-effects play an important role throughout the duration of the treatment. Some participants expressed a need for more information. | “I would like a bit more information really, because of this gap there is in terms of the long-term implications of the treatment and whether I should be taking it or whether I shouldn’t.” Interview 2  “I wished it had been explained a little bit more, with the side-effects of them. What to expect and also, whether there was any other options of drugs that I could have taken.” Interview 6 |
| Some of the women were always looking out for the latest information in the field. Asked about their main source of information, their answers varied. While some referenced their healthcare providers, others mentioned google, specialized websites and friends as their main sources of knowledge. | “I suppose it was the internet, really, and obviously talking to, if I wanted to I could have spoken to a professional. I did consult with somebody who is a pharmacist, but more as a friend.” Interview 1  “It would be my GP now. Obviously, when I was under the hospital, which was for the first five years, then it would have been my consultant at the hospital.” Interview 2  “I have to say, I did do quite a lot of research online. I’m a researcher, although I’m in arts and humanities, but I did do a lot of online research.” Interview 4  “I went to Doctor Google. And then having at times, if I’d read too much nonsense on Google, I then got in touch with either Macmillan or Breast Cancer Now.” Interview 11 |
| While the internet proved useful to most women, others talked of the dangers of seeking information online, because much of the information they were able to find came from women who were experiencing difficulties with the treatment, which some of the participants found alarming. | “You have to be a bit careful though, because it can be a little bit frightening. So, I think you have to pick and choose the information that you actually take from that, very carefully, but yeah, but, a lot of the time, I try not to think about it.” Interview 6  “I think for somebody who’s a medical professional, I think yes, because you can weed out the incorrect information, but I think for somebody who isn’t, I think it could be a big problem that they can find incorrect information on the internet or things that might scare them.” Interview 7  “I Googled things and I knew what to ignore and what I thought was good. So I, you Google things and all these things come up and all these chatlines come up with everything, people, with all the negatives, because no one goes on a chatline and says how good it is, it’s always what goes wrong. So I learnt to ignore most of that and go onto Cancer Research or Breast Cancer Now sites and look at the information there.” Interview 12 |
| However, some women talked about their experience of visiting specialized websites rather than forums and how this helped them find the information they were looking for or guided them to other possible sources of information and contacts. | “I went to breast cancer, there is a website breast cancer and then there is another one, the Macmillan one.” Interview 8  “If I’d read too much nonsense on Google, I then got in touch with either Macmillan or Breast Cancer Now. I’d ring them up and ask them.” Interview 11  “I learnt to ignore most of that and go on to Cancer Research or Breast Cancer Now sites and look at the information there.” Interview 12 |
| One participant reported that despite having access to the information she needed online, she was not told by her healthcare provider where or how to find it. She felt that this responsibility lay with the medical team and that they should have explained it better. | “I had access to information, if I needed it I knew where to look, but I didn’t feel that was offered. I often had to ask for it or look for it.” Interview 1 |
| Another participant talked about how lost she felt, not knowing who to ask or reach out to when in need of information. She felt that everything was rushed initially and that, once she had begun the treatment, primary healthcare providers were reluctant to discuss a medication they had not prescribed, leaving her feeling abandoned. | “I didn’t really know who to ask because as I say, the oncology team are quite rushed, and you’ve gone through your treatment, so you just feel as if they’re giving you them and then my doctors aren’t really that helpful with it because they’ve not really prescribed it. So, I think you’re in a bit of no man’s land in a little way where the hospital, the oncologist is dealing with life and death not hormone treatment and your doctor’s thinking, well actually I haven’t prescribed that, that’s the hospitals, so yeah you are a little bit out on your own, I think, with them.” Interview 3 |
| Differences between participants and their need for knowledge emerged from the interviews. Some were keen to have information in advance to help them deal with issues should they arise. Others preferred not to know about possible issues until they encountered them, fearing that if they knew in advance, for example about the drawbacks of hormone therapy, they might worry more and allow their ‘imagination to run wild’. One participant shared that she found herself experiencing both these scenarios during different phases of her treatment. | “I prefer to know, yes, and I find it reassuring to know.” Interview 2  “I think the trouble with telling me about side-effects is I might find I have them where if I know that I could have them, if that makes sense.” Interview 12  “Not always. Because the more you know, the more worried you are, to be honest.” Interview 8  “I go through different phases of that.” Interview 4 |
| One participant talked about the necessity of knowing what side-effects she might encounter, and how best to cope with them. She mentioned the need to stay positive, but in a realistic manner: to her, positivity and realism needed to go hand in hand. | “Yes and also the positivity in a realistic sort of way, because I've spoken to people since and there’s people going through chemo it’s all right saying it was fine, but it’s not fine, so taking letrozole I, if people ask me about letrozole and tamoxifen I’ll say it’s fine, but I have had problems with different brands and I do get quite stiff in the morning, but if I walk it gets much better; so it’s knowing the negatives but also working out how to make that better, not just knowing the negatives. So I think being informed about what could happen but what could make it better, yeah, is good.” Interview 12 |
| Side-effects of the treatment |  |
| The side-effects of hormone therapy treatment are one of the most important factors associated with taking the medication. The participants in this study experienced side-effects very individually, some reporting minimal to no side-effects, others moderate to severe. Those suffering minimal side-effects described a better experience with the treatment. | “It was fine actually, I didn’t have any side-effects.” Interview 1  “I would say that the side-effects have not been so drastic that it has massively affected my quality of life. I would say that, also having taken it now for four years, I would say that I’m less aware of the side-effects.” Interview 4  “I think when I started I didn’t know how bad the hot flushes would be and they haven’t been as bad as I know, I have a good friend who’s really had horrendous hot flushes with Tamoxifen and had to come off it in the end. So I think from that point of view I was quite pleased that it was maybe not as bad as I thought.” Interview 7  “No side-effects really. Not something that I noticed for example, something that changed in my routine, no.” Interview 8 |
| By contrast, the participants who reported moderate to severe side-effects described a worse experience on the treatment. | “Yes, I don’t like taking them to be honest. My hair was thinning, my nails were really weak, and the joint pains and you read all the side-effects and I do think, you do get the side-effects. I just feel as if I’m being pushed into old age before it’s my time, that’s how I feel.” Interview 3  “I’ve experienced quite a few side-effects from the drug, which some days, I really struggle with, so, I’ve put on quite a bit of weight since I’ve been on it and I get a lot of pain in my joints.” Interview 6 |
| The reported side-effects were both physical and psychological in nature. The physical effects mentioned by participants included hot flushes, headaches, muscle pain, joint pain, lack of energy, drowsiness, blurred vision, dry mouth, vaginal problems (dryness, soreness and continuous infections), nausea, weight gain, fluid retention, thinning hair, weak nails and impaired memory. The psychological side-effects reported by some participants included anxiety, negative body image and insomnia. | “I would say that at first I felt, I definitely had some side-effects, and I’d say that those probably continue. The main thing I would say is anxiety levels, I think, obviously there’s a bit of hair thinning, all those kind of things. So, I don’t enjoy taking it.” Interview 4  “So, the hot flushes I’ve had quite, I say not badly, they, they’re a pain. Lots of musculoskeletal tightness. I don’t sleep very well. Memory, I’m sure it’s made my memory a lot worse. I have vaginal soreness.” Interview 7  “The worst one of all was very severe headache, crippling, so I could barely move. What comes with the headache is, can't concentrate, can't think, can’t function, barely function. I had, feeling sick and that, that I can deal with. Balance, I have a bit of vertigo, but the loss of balance was bad and I fell twice at home because of it; nobody did anything about that, I reported it. Muscle pain and pain. Feeling, just no energy, very severe urine infections, very severe. I had some of the others, swelling and things like that. Oh with the headache the most awful irritability, as if there was wire wool inside my skull, and that was exhausting, not to really have a go, and it wasn’t related to anything that was happening to me. It was really, really bad. Blurred vision, I have dry eye syndrome anyway, so that got worse. Dry mouth, that didn't trouble me, I could put up with that. More of arthritis. Very thirsty. Vaginal infections. Just feeling very weak, very weak and very difficult to function and plan anything, very difficult. Feeling sick, not often being sick, weight gain, I didn’t mind about that. Fluid retention, joint pain, muscle pain. All of that I could put up with but it was the headache and very sleepy. Couldn’t stay awake sometimes and tired. It’s very difficult to, even doing nice things, it’s very hard to do that.” Interview 14 |
| The severity of the side-effects could reach the level of being disabling, some participants reporting difficulty in carrying out their normal daily activities. | “I took the tamoxifen, I think about a year, but unfortunately I had just about every symptom on the list. It was just awful. In fact I had double vision, I had terrible sickness, all, I mean all sorts of things. In fact so much so they actually gave me a brain scan because they thought maybe it had metastasized into my brain. So, yeah. So obviously following the spec when I, once I’d, they’d done me the brain scan and they’d realized it wasn’t they realized it was the tamoxifen.” Interview 13  “I've had viral encephalitis in the past, very badly, and it wasn’t diagnosed at the time so I was ill for a long time, and the headache from that is, well it’s as if your skull has shrunk. And I would put the headache from, that I had from letrozole, not as severe as that but in the same class. That’s like a slight movement of the head is just and sometimes all I could do was just lie completely still.” Interview 14 |
| I asked the participants whether the side-effects were as they had expected or if they came as a surprise. Although some said they were as expected, others shared their shock about how severe some of the side-effects proved. In some cases, even if the woman knew in advance about the side-effect in question, its severity came as a complete surprise to them. | “No, because I’d read very clearly the instructions, so I was expecting them.” Interview 9  “I am surprised by that tiny little white tablet, what it can do to your joints and, people laugh at hot flushes, but they can be really, my sleep pattern at night is dreadful − it’s off, on, off, on, and you’re tired, I feel tired.” Interview 3  “I was surprised at how, you know, volcanic they were where all of a sudden you’re sort of, I don’t know, kind of sat minding your own business and then all of a sudden you get this rush of fire coming up through your body and frazzling your head.” Interview 11 |
| A side-effect of the treatment mentioned by one participant was the effect hormone therapy can have on intimate relations with a partner or significant other – an issue the woman described as “quite a challenge”. | “I suppose if you think what an impact it does have, it does have an impact on everything. Even my, with my husband, the intimacy with my husband it’s affected that. You don’t really talk about it because you just think, oh well, but we’re still quite young and to be going through it now it’s quite a challenge really, yeah.” Interview 3 |
| Women reported difficulties with the body changes caused by the treatment and with having to adapt to looking different. Weight gain was reported multiple times as a source of distress, another factor being the effect hormone therapy can have on hair colour and thickness. These changes were described as “difficult” and “depressing”. | “Oh yes, because I looked completely different. I still look different. I have grey hair, I had brown hair before, I lost my hair and it’s come back grey. So I looked completely different. So it’s finding yourself as well, so I think that’s not typically, not really the hormone treatment, but you're taking hormone treatment when you look different from the other treatments and that’s quite difficult.” Interview 12  “My hair has changed completely and that’s my, that’s the thing that depresses me most or upsets me most. So, and it’s like baby hair now and it’s so thin compared to, I had a lot of very thick hair which came back after chemo but the anastrozole has definitely affected my hair.” Interview 9 |
| Another issue reported was the loss of control while on the treatment. One participant talked about how the treatment pushed her into premature menopause. The sudden transition from being a healthy young woman into someone suffering from menopause-like symptoms was difficult to comprehend and adapt to. | “Obviously, you go through the menopause, you’re getting all the signs of being menopausal at quite an early age and no one really explains all of those properly to you, I don’t think, because you just stop bleeding and then that’s it. So, you haven’t gradually, it hasn’t gradually happened, so all of a, you’ve got none of that. So, the hair, the dry skin, vaginal dryness, all of that comes in and you’re just like, woah I didn’t expect all this. So, it’s quite a sudden transition from being a healthy 48-year-old to feeling that you haven’t got much control anymore.” Interview 3 |
| One of the disturbing consequences of the treatment is the effect it can have on memory. Multiple participants used the term “chemo brain”. However, from the participants’ point of view, the matter is not always straightforward. Participants questioned whether the effect on their memory was attributable to their chemotherapy, their hormone treatment, or simply to getting older. One participant, asked about whether she ever forgot to take the treatment, identified the stress that goes with the treatment and continues after finishing it, as the reason behind her memory worsening. | “I’d also say that definitely, I can’t hold things in my head the way I used to. Now I don’t know whether that’s chemo brain, but it’s four years since I had chemo. I don’t know whether it’s age, I’m already in my sixties, or whether it has something to do with the medication, but definitely I would say I am forgetting things.” Interview 4  “I also think that I don't know whether it’s chemotherapy, I don’t know what it is, with what I've been through, I don’t know if it’s the letrozole, but my mind certainly can't take in as many things and work on all of them at once.” Interview 12  “Oh God, yes. [Laughter.] You’ve seen what my memory’s like and I’m 17 years on. During, I mean they talk about chemo brain, which maybe if you’ve spoken to other ladies it’s come up once or twice. I didn’t do chemo but I really had a problem with chemo brain after my treatment and they suggested that it’s not actually necessarily the drugs. The reason why people like me, who didn’t do chemo, still have trouble with it is that in fact it’s the stress level. The stress of being diagnosed, the stress of waiting to have your treatment, the stress of doing your treatment, the stress of everything that goes with that. And then afterwards you would expect, well, that’s all that stress gone away, you’ve not got cancer any more, and the stress then of wondering will it come back. So that basically is where the, where the, where it all comes from.” Interview 13 |
| Participants mentioned their desire to feel like their usual selves again and regain the sense of normalcy in their lives. One participant talked about the fact that she was looking forward to stopping the treatment, because taking a pill every day was a constant reminder that she suffered from cancer, and about how this feeling acted as a barrier preventing her from feeling normal. Another woman spoke of her desire to return to her old self, before the cancer diagnosis, and to go back to work as though nothing had happened; a third talked about her desperation to find a way to manage her side-effects, just to be able to live “something of a normal life”, as she described it. | “There is that sort of psychological effect where when you stop, when you do stop, and I’m talking about at the very end stage when you stop taking it, you do feel OK, so you can sort of, you’re obviously starting to normality that you’re no longer, you associate taking medication with something wrong with you, so it’s like progress” Interview 1  “What I do think, and I think this is really, really important, is that I did, I wanted to go back to work as soon as possible. I thought that I could just take up my life as if nothing had happened, and that is not the case.” Interview 4  “Because I thought that if there was information about what caused it then maybe might be able to look or reduce it to manageable levels, I wasn’t so naïve that I thought they're going to be able to take away all these effects, but maybe I can go on living something of a normal life. Maybe I have to reduce activities or, but maybe I can feel more human than I do at the moment.” Interview 14 |
| Despite their wanting to feel normal again, going back to work was emphasized by some women as being a difficult step to take. One participant mentioned returning to work for financial reasons, talking about trying to go back as though nothing had happened and how she now thought this a mistake she should have avoided, but due to financial constraints and previous work commitments had felt forced into. Another woman talked about having to go back to work, despite feeling really sick, due to her commitments to others and a feeling of needing something else to occupy her. | “It had just taken so much of a toll in every way, physically, emotionally, psychologically, that looking back on it I think I probably should have done a phased return to work. But of course, there were financial implications, because you can take six months on full pay, and then after that it’s half pay, and I have a mortgage to pay. And that was partly why I did the flexible working, so that that could go against the sick leave period. When you have PhD students, the support you give to them, you can’t measure that in hours, actually. And also we’re quite a small department […] quite a young team. So I see mentoring responsibilities as being quite important there, plus, of course, my own research. So it’s very difficult for that not to take up all your time, and that’s something I’m working with, is that idea of really trying to pull back.” Interview 4  “I had a PhD student and I thought, I was very positive the whole time, it was just reflecting back I think, oh gosh. It’s strange but I kept working even though I was really sick in the morning. I would, when I was at home, I would make sure that I was dressed, and I would get to my desk by about 11. I just needed to have something else to think about and so and even when I was in hospital.” Interview 9 |
| Some interviewees’ felt that the treatment and its side-effects had a negative influence on their social life. One woman talked about how, since being on hormone therapy, drinking caffeinated beverages or alcoholic drinks had caused her to immediately experience a hot flush. Another participant talked about the fact that since beginning the treatment, and due to its side-effects, she had tended to be more bad-tempered. | “As I say, my health, and even to the stage where if you go out for a drink now, I’m always thinking, shall I have coffee with caffeine or an alcoholic drink, because that just gives you a flush immediately.” Interview 3  “Definitely, yeah. And, sometimes it can make you grumpy, if you’re, if my joints hurt for a few days, it gets on top of you.” Interview 6 |
| Not everyone reported the same experience. One participant shared how the treatment had influenced her social activities at the start, but no longer did to the same extent. Other participants, who experienced minimal side-effects from the treatment, said that it did not affect their social lives at all. | “It did for a while, it doesn’t so much now but it did for a while, but we live in a quite isolated place, so we don’t have a huge social life.” Interview 9  “I don’t think, because I didn’t have many side-effects.” Interview 2  “No, I wouldn’t say that, it’s done, it’s particularly done that. I’m not, I’m not off to parties every evening.” Interview 4 |
| Participants report their difficulty in distinguishing whether the side-effects they felt were hormone treatment-related or caused by other treatments or simply getting older. Hormone treatment is long-term, so eventually perceptions about side-effects tend to become entangled with other things, some participants saying they found it difficult to know what was causing what. | “Well I’d had a few joint pains and aches and pains but then I thought that was, that could have been anything at my age whatever.” Interview 1  “I have, I say lots of musculoskeletal tightness, the problem is you have these symptoms and you don’t know whether they are due to the drug or if you would be like that anyway.” Interview 7  “You know, it’s difficult isn’t it, because you kind of think, well, if I’d not taken the anastrozole, would I have had them or would that be my menopausal journey, was that, you know…” Interview 11 |
| The side-effects of hormone therapy were not the only concern participants shared about their medical treatment. Another was a fear of side-effects from other treatments taken to manage those side-effects. One participant expressed concern about co-codamol, an analgesic she was taking daily. Another participant, prescribed an anti-inflammatory medication to take daily, reported that due to her fear of the long-term implications of that medication, she had decided to reject the prescription. | “To take co-codamol every night, surely that’s not that good because when they tell you, the pharmacist always says, oh you shouldn’t be taking it for more than three days or so, and you think, well actually they prescribe it to me to take every night.” Interview 3  “So I then went to the GP and the GP then said if I took anti-inflammatories for ten, for five years. Well, I read The Lancet, same as he does, so I told him that and said, no, I don’t think so, thank you very much indeed. I really don’t want to do that because the statistics on anti-inflammatories are people die, 5,000 people a year die taking them long term. And so my choice is do I die of cancer, do I die of taking anti-inflammatories? I’ll take my chance with the Arimidex, quite honestly.” Interview 13 |
| Side-effect management |  |
| Participants talked about side-effects management and techniques they implemented to make their experience better. Side-effects management is a big part of living with a hormone treatment. One of the mentioned ways of managing the treatment’s side-effects was to take other medications. | “They put me on Venlafaxine, which they said is an antidepressant, but they found that it helps with hot flushes. My concern was that I’m somebody that’s quite happy naturally, and I didn’t want to take something that, when I came off it, might make me feel different, when normally I’m a happy person − yeah, even with cancer I’m a happy person. They said no, you don’t need to worry because it’s quite a low dose, so I went on 75mgs and it certainly didn’t eradicate the hot flushes, they were still debilitating, but I carried on with that. Then I decided I’d come off it because I wasn’t sure that it was really helping, and then when I did come off it I realized that actually the hot flushes were even worse without it, so it had; although it didn’t get rid of them and they were still severe, it was even worse if I didn’t take it.” Interview 5 |
| Hormone therapy can negatively affect bone density and lead to the development of osteoporosis. Some participants talked about having to monitoring their bone density throughout the treatment and having to take alendronic acid to improve their bone health. | “My consultant wrote a letter and then I had a letter from the GP saying, yes, they’d arrange for me to have a bone density scan. And then I found that I’d osteopenia and so they prescribed alendronic acid for that, so I was taking that at the same time as taking the Arimidex, yeah.” Interview 1  “I should also say, because this is part of the whole pharmaceutical package that I’m on, I also have osteoporosis. So that is a side-effect of Anastrozole, which I’m on. So I also have to take alendronic acid, which is an awful nuisance. But I can see that, I’ve had a couple of bone scans since then, and the bone density is increasing.” Interview 4 |
| One of the lifestyle modifications mentioned by some participants was either working fewer hours or quitting work completely. | “I went part-time, at work. So, that’s what I mean when I say I made lifestyle adjustments.” Interview 4  “Well I've given up work, which is, I took early retirement because I was off and then realized my job was quite stressful and family became more important than working all hours.” Interview 12 |
| Some participants talked about wearing multiple layers to counter their sudden hot flushes; this way they could adjust what they wore by taking a layer or two off when the felt a hot flush coming on and put them back on when they started to feel cold. One participant talked about her use of humour as a coping mechanism if experiencing such an episode when with friends or colleagues. | “Yes, I, where, I was always a cold person and then when I was on that I couldn’t wear anything that, for warmth, because as soon as I put on something that I would have worn before [Nonverbal sound] hot? Yeah, so yes, I had to change the way I dress dramatically, yeah. Then of course when I had the hot flushes with the Tamoxifen, that was just more of a humorous thing because I was always taking, it was a joke. Because they, I think although I had a serious cancer, because of the way I coped with it, people, they forgot really quickly that I’d even had it. If you, that’s what it was like, yeah.” Interview 5 |
| Diet and exercise are a common method of adaption and something multiple participants talked about. One participant reported giving up smoking, another deciding to drink less alcohol and cut down on red meat. | “I’ve kind of thought about my diet a bit more. I gave up smoking. I suppose that my adaptions were wearing lighter clothes, because I felt hot all the time. I don’t wear tights anymore. I very rarely wear woolly jumpers, even in the winter, so those are the adaptions I’ve made to it, but I try and see it as a, actually it’s had a benefit because it costs me less money heating the house.” Interview 11  “The other thing I would do is that I, I don’t do a lot of exercise, but I do a lot of stretching exercises and I walk, so I’m trying to do more exercise. So more exercise, less alcohol, I try to watch what I eat so that we eat more healthily, I’ve cut down on red meat, that kind of thing, and green tea or other herb teas and turmeric. That’s probably about it.” Interview 4 |
| One participant talked about Pilates as a useful exercise for countering joint pain and stiffness, another about walking being a helpful technique to reduce joint stiffness. | “I do Pilates every day, every morning and that really helps with knees because I have aches in my knees sometimes.” Interview 9  “Yes, I think it would. I think knowing that if you’ve got joint stiffness, it’s quite painful, so the fact that you go for a walk might not be the first thing on your mind, because the first thing on your mind would be sit down and have a cup of tea. But actually a walk, walking does help it and exercise helps and I'm not very good at that, but it does, so…” Interview 12 |
| Meditation and yoga were also mentioned by one participant as useful techniques for reducing stress and improving overall health. | “Yeah, I do meditation, mindfulness, yeah, so yoga, meditation tapes where you just hear and they tell you how to relax and things like that.” Interview 3 |
| One of the techniques mentioned by participants as a way of countering side-effects was taking the treatment at night rather than in the morning. | “So yes, so I started taking them, had side-effects, especially the sickness side of them, and the breast cancer nurse just said to take them at night or just to change when I took them, so I take them at night now instead of in the morning and to be honest, the side-effects have worn off, the sickness side but the hot flushes haven’t but that’s another, yes.” Interview 3  “Yeah, and it’s possibly because I could take them at night but then I prefer, I just think I’m fine with taking them at night and I’d rather not have that in the daytime, so yeah.” Interview 9 |
| However, this did not work for everyone. One participant shared her experience of taking the treatment at night and how it affected her sleep to such a degree that she had decided to go back to her previous routine of taking it in the morning. | “I did think, I think we were on holiday actually, and I thought oh I, I'd spoken to somebody and they said they took it at night, so I thought I’ll, why don’t I take it at night because then if I'm sleeping I might not notice the aches, but then I couldn’t sleep because of it, I ached so much in the night, whereas I think during the day, because I have to keep going I don’t notice. So then I went back to take it in the morning again, so…” Interview 10 |
| Some participants experimented with various alternative treatments for the management of their side-effects. | “I’d have to say I take green tea, I take turmeric, but, and I think those things are useful, but I’m not sure that they actually outweigh the benefits of pharmaceutical medication.” Interview 4  “My day job if you like is natural health products and I took an aloe vera-based drink with glucosamine and chondroitin and MSM in it, and to be honest with you, within two weeks I was like a new woman, it was amazing because I’d been suffering with really bad shoulder pain, knees, ankles. I couldn’t get up off the floor it was so painful, and no strength. So, and once I’d taken, started to take this product, I actually, I was, two weeks and I was, as I say I was like a new woman, it was amazing.” Interview 13  “I went to this medical herbalist who was recommended, she has a good reputation in Oxford. What she gave me eased the headache and improved my concentration, it still wasn’t any, wasn’t good, but and she gave me for the vaginal issue, she gave me, because all the other creams and stuff they helped a bit, she gave me comfrey oil – fantastic, and the vaginal infections cleared. The urinary infections I had very, far fewer of them, to the extent that my GP wanted to know what herbs were, oh can you tell me, I have women coming who can't, and so for me that made it a little more bearable.” Interview 14 |
| However, some participants showed reluctance to use alternative treatments, fearing their possible side-effects. | “I wouldn’t have considered taking any sort of alternative medicine because I knew there may be reasons why you shouldn’t do that.” Interview 1  “No, because I considered sort of like the herbal things, like red clover and black cohosh, and there’s so many bad reports of liver failure and all this sort of thing, I thought it’s just not worth it.” Interview 7  “I thought about it briefly and then I haven't. And I don’t know why I haven’t because I know alternative treatments do work for some people, but I'm living my life as I did before, really.” Interview 12 |
| Changing the treatment from one hormonal medication to another was not uncommon. Some women were advised to change their medication by their healthcare providers. Some decided to change it by themselves, either to escape the side-effects of the treatment or owing to a preference for one treatment over another. | “The reason he, he showed statistics, it was a different consultant and he queried why I was on tamoxifen so I explained and he showed me the statistics to say that the chance of reoccurrence was less at my age, so post-menopausal, if I took letrozole. He said tamoxifen worked better with pre-menopausal ladies, so what he did was, he showed me the statistics and looking at it, even though it was a very small percentage, that meant I would have less chance of reoccurrence that, even if it’s 1 in a 1,000 better, that, to me, that was worth changing the tablets. So and he also put me on some calcium and vitamin D for my bones, so that took away the worry of osteoporosis as well, so…” Interview 12  “I took the tamoxifen I think about a year but unfortunately I had just about every symptom on the list. It was just awful. In fact I had double vision, I had terrible sickness, all, I mean all sorts of things. In fact so much so they actually gave me a brain scan because they thought maybe it had metastasized into my brain. So, yeah. So obviously following the spec when I, once I’d, they’d done me the brain scan and they’d realized it wasn’t, they realized it was the tamoxifen, they actually did prescribe with the Arimidex.” Interview 13  “I got the prescription for the tamoxifen but I investigated myself the possibility of transferring to letrozole.” Interview 14 |
| Some women decided to stay on the treatment rather than change it, fearing that doing so might affect them negatively and cause them to experience other side-effects. | “Even though potentially I could have less hot flushes, anyway. So, in a way it was sort of a blessing in disguise that I would prefer to stay on the Tamoxifen than change to something that I wasn’t really sure about.” Interview 7 |
| One of the techniques mentioned by participants to counter the side-effects of the treatment is to change the brand of the medication. The reported experience shows a clear difference in some cases where participants report having a better experience on one brand of the treatment than the other. The idea of changing the brand of the treatment in mentioned by one participant as something that is not usually discussed in the consultation visit. | “Although I don’t know if the other, anybody else has said this, there are different types, there are different manufacturers of these tablets and with one manufacturer it, I had very, I had really stiff muscles, bones, my ankles were really sore and I've found that if I stick to certain manufacturers of letrozole I am much better. So if I, I now put on my prescription, I do not want this brand of letrozole and I don’t know. Yeah I have told somebody else this as well, her elderly mother took some and she asked for a different type as well and since the side-effects seem less. So I don’t, it’s just one of these things, it might just be, I don’t know, no but it definitely, yeah.” Interview 12  “I do obviously know but there are different generic, there are different manufacturers and they’re not necessarily exactly the same and so my suggestion if they’re having problems would generally be if you feel that you want to give up, first of all I would reassure them that it’s not normal whatever it is they’re doing, that they’re getting if it is, and then I would suggest that if they suggest they don’t want to come off it, they want to come off it I will obviously tell them about the benefits of it. But also I will say to them to go back to their GP or their consultant depending on which stage they’re at to actually suggest that they perhaps take a different type because I’ve had quite a few people that have gone from one to another and found that one suits them so much better. So that’s not something, again that’s not something that’s usually mentioned during a consultation. So it’s quite important really.” Interview 13 |
| However, not everyone shared a similar thought process. One participant wondered if the idea of changing the brand of the treatment and having a more positive experience on one brand than the other is something that is just ‘in patients heads’. She thought that as long as both have the same amount of the active ingredient trying a different brand would not actually make a difference. | “I mean I did do a search once and it had women saying that they, their, and in fact, I think Breast Cancer Care don’t pooh-pooh this, but it depended on the brand of Tamoxifen they were having, depending on how their symptoms were and you’re thinking, well either that brand isn’t worth, it’s not by available or, it’s all in their head, so it’s things like that you sort of think, well I don’t, unless that brand’s not giving you the amount of Tamoxifen it should be, then it’s, it’s obviously not going to make any difference.” Interview 7 |
| In an attempt to manage the side-effects of the treatment one participant talked about her experience with a severe headache throughout the duration of the treatment. Due to the severity and after exhausting all standard solutions she tried to identify the reasons and mechanism of action of the side-effect to try and counter it by contacting the manufacturing company of the medication. She was told that the mechanism of action that causes the severe headache was unknown to the company. | “The other thing I did do was I phoned the company that makes letrozole, is it Fem, well that’s a trade name isn't it, anyway that company. Because they had a customer helpline and I said, I'm wondering if you know what the mechanism is for causing the headache and the person on the end of the lien said, oh we’re not allowed to give out that kind of information and she wasn’t rude, she was a bit abrupt but I think she was out of her depth. I think she just didn’t know how to handle the and I'm on the other end of the line thinking, please don’t speak like that because I am very irritable and I am really struggling not to shout at you down the phone, I'm really struggling to be really polite and I feel as if I've got something clawing inside my head. And no, no we’re not allowed to give out that kind of information. Why? Oh no, no, we’re not allowed to. And then I said, oh I think she said something along the lines of, it might upset people or it might, I, she didn’t know what to say basically and then I said, well I am a healthcare professional, I think I can deal with the information and she said, are you a doctor, I said no, I'm a nurse, oh. Well I’ll see if I can put you through to somebody else. And actually I think somebody phoned me back, who did know all about it, was involved in the actual production of the medication and she said, I'm sorry we don’t know.” Participant 14 |
| One participant, describing herself as a “glass half full” person, decided to see the positives in the side-effects she was experiencing, thinking of her hot flushes as a way of saving on heating bills in winter. This determination to use optimism and banish pessimism was one way of dealing with her side-effects. | “But the benefit of the anastrozole was that I was no longer cold. I’m a hot, hot totty now. I’m a hot bird. So, the benefits for me is it doesn’t cost me as much in heating the house, even in the winter, because I’m hot, whereas prior to the menopause, I was a, I always felt cold, so I’d have the heating on, I’d have jumpers on. Now, I’m just too hot. So my quilt, for example, whether it’s winter or summer, is 1.5 tog and I’m still too hot with that at times, especially in the summer. Whereas before, I’d be tucked up under a 15-tog quilt feeling cold. So, for me, in a funny sort of way, it’s been a bonus. I’ve saved money.” Interview 11 |
| Adherence |  |
| Participants shared how they would usually try their best to adhere to the treatment as prescribed, making the treatment part of their daily routine to make sure they never forget to take it. One participant shared her experience with pill boxes and how she found them a helpful aid in adhering to the treatment. | “I very rarely miss it, and I take it at the same time every day. So I take it with breakfast, and that works.” Interview 4  “Well I take blood pressure tablets as well, so every morning when I'm in bed I have a glass of water at the side of my bed and before I do anything I take my two blood pressure tablets and my anastrozole, first thing in the morning.” Interview 10  “I bought myself a Monday to Friday pill box and I’d put all the pills in it on a Sunday. So, it ran out on a Sunday and then I’d refill it for the rest of the week and then it was kind of like on my kitchen side near the kettle. So the first thing I went to in the morning would be the kettle, and next to the kettle was my pill box, so I’ve got no excuse not to, no to forget.” Interview 11 |
| When asked whether they ever forgot taking their medication, most replied negatively. The only time participants reported doing so was when a change occurred in their daily routine. So things like waking up late, having a meeting earlier than usual, or being on the move, were reported as reasons some forgot to take the treatment. | “But I always take the Anastrozole with my breakfast, so I rarely forget actually. It has happened, I only forget really if my routine is completely out of kilter. In those cases, if there’s something I have to deal with in the morning and things are not quite, or I’m up later than usual, it’s something that, those cases I might forget, but I adhere very strictly, I’d say.” Interview 4  “To be honest, it doesn’t, I just, it’s just routine, it’s now routine. I have it in a medicine box, I think I've forgotten it maybe in the past two years, three or four times, but it literally is because I've gone away for a night and forgotten my tablets or, it’s not that I don't want to take it it’s just that it slipped my mind.” Interview 12 |
| Asked how they felt about forgetting to take the treatment, participants generally did not worry and didn’t consider missing it the odd time to be a big deal or something they needed to worry about. One participant did share how forgetting to take the medication made her feel “anxious” in the early days of the treatment, but added that she would not worry about it so much now. | “Oh, it’s happened so rarely. I reckon after four years of taking the thing, if I forget it’s not going to make that much of a difference really, so I don’t bother. I’ve never forgotten to take it for a week or something, you know, so…” Interview 4  “Well first time I did it, I thought, oh my goodness, I need to, what do I do? And then I realized that actually, they're quite strong, so I didn’t want to take it when I got home at night and then take another one in the morning, so I just missed a dose. And I just think that if you're taking tablets long term and one dose gets missed, then your body soon adapts to the next dose, and I just, I didn’t, I don’t think I even looked it up. I think just common sense said to me, look don’t panic, this is long term, you take it every day, one tablet is not going to affect you too much.” Interview 12  “Yeah, I was anxious, I was really anxious. But it was a long time ago, and I would be less anxious now.” Interview 9 |
| Participants revealed a range of views on the topic of drug holidays. When asked whether they had thought of taking one, some participants talked about how terrifying the idea seemed to them, fearing recurrence so much that the idea hardly struck them as an attractive option. | “I would be too frightened that something might happen in that period that I wasn’t taking it.” Interview 6  “No. No, I'd be too scared in case the oestrogen came back and caused a problem. No, no, I just take it religiously.” Interview 10 |
| But not everyone shared this view. Some participants talked about their interest in pursuing the idea of a drug holiday and their desire to discuss it with their healthcare provider. One participant talked about how some of women from her support group had taken a short break from the treatment on their own initiative. Another talked about her experience of taking a break from the treatment in the hope of countering some of the hormone therapy’s side-effects, before deciding to go back on it after a month had passed without signs of improvement. | “I also would be quite interested to talk to him about taking a break, for example maybe taking three months off it to see what, how I feel, whether that makes any difference.” Interview 4  “I’ve had ladies in the group that have been through the group that have done that and simply really themselves they’ve said, look, I can’t take this anymore, I’m just going to come off it − and then they’ve gone back on it again.” Interview 13  “I did come off it for a month, I think that was to see whether my stiffness improved, but it didn’t seem to make any difference, but then whether a month was long enough I don’t know.” Interview 7 |
| One of the things participants spoke of in relation to hormone therapy adherence was the fear some had of regret and of having no one but themselves to blame was their cancer to return. Their anticipation of regret was a driving force making some participants more adherent. | “No statistics tell me that this is doing the best it can for me. And I just think I would never forgive myself if I took a break and my cancer came back, I would be convinced it was my own fault. Whereas this way if it comes back I've done the best I can.” Interview 12 |
| One thing participants were understandably keen to learn was whether their treatment was working or not, not least because a positive answer to the question could provide added motivation to keep taking it and accommodate any side-effects. One participant talked about how, when she did her annual blood test for hypothyroidism, the results showed that her oestrogen level could not be measured, convincing her that the treatment was working and doing what it was supposed to do. She believed this test should be offered to all breast cancer survivors on hormone therapy treatment in order to ease their minds and improve their experience. | “I think part of me thinks how do I know they're working, that’s the only query I have. If somebody could give me a blood test and just say no, you haven’t got any of those hormones in your body, I think I would feel quite reassured, do you know what I mean? It’s kind of, you take these tablets, it doesn’t seem to affect me very much, so what is it doing?” Interview 12  “The other thing that kept me going was, I have thyroxin, which is prescribed privately because I was never followed up in general practice. And when my blood’s tested annually, they do a hormone assay as well, and I could see that they couldn’t measure the oestrogen, it wasn’t measurable, and I thought, well, the letrozole’s working. I thought, well I'm not going through this hell for nothing. I know that it is blocking the oestrogen because they can't even measure it and it’s below the normal; maybe there are very refined tests that they could do, but with the normal ones it was below. This is because I, when they, at the clinic where I get the thyroxin from the blood test automatically that you pay for, checks all of them, so I had that information. I think actually, if I hadn’t been having that, it would have been very useful to be, have been offered that, to at least know whether it was doing something I think really.” Interview 14 |
| One reason participants gave for not adhering to the treatment was having other priorities. When asked if she would ever consider not taking the treatment, one participant shared that she would actually stop if she decided to have another child. She thought having another child would prove a higher priority than her treatment, driving her to discontinue it. | “Yes, if I was about to have another child. But if not, then no, I don’t think so. I normally comply with what they tell me to do, to be honest, because other people they have more evidence on how to treat stuff, so I say, OK fine, I’m not an expert in this area so I follow instructions.” Interview 8 |
| The experience of having the prescription dispensed differed from one participant to another. One of the issues mentioned regarding obtaining the medication was having to do it on a monthly basis. This was found to be frustrating, leading some participants to question why they could not be given it in longer-duration batches. One participant mentioned the effect being limited to a one-month supply had on her adherence level, as she missed some doses of her treatment due to running out of the medication. | “I did have some problems initially with the regularity of prescription. It’s, you could only have a month at a time, and I knew I was going to be on this treatment long-term, so why could I not have six months’ worth and if I was going away on holiday just trying to get enough to cover. You had to plan ahead to get, make sure you had enough prescription to cover and things like that.” Interview 1  “The thing that is a nuisance is actually having to pick it up every month from the pharmacy, although it’s not that far away, and timing that to the, when the next batch starts. So I’ve sometimes, again very rarely, lost a day or something between the starting of one in there.” Interview 4 |
| One participant talked about her experience of obtaining the prescription monthly and how she agreed with her doctor to change to having a two-month supply instead. She also talked about how taking this step helped her adhere better and think less about the medication and cancer. | “The other thing was the doctor initially said that she only wanted me to have a month’s supply, but I just, in the end I said to her, I'm on this for seven years, do I really have to apply every month? So I now get two months’ supply of tablets, which works better. A month, I was continually running out, the month goes by quickly, so again I would, unless I'd have asked, I would be going every month. So again, I just think people should know that you can get two months’ supply of it, because it saves you thinking about it all the time. To have to think about your tablets every month just brings it back to your mind doesn’t it?” Interview 12 |
| Participants ordered their prescription online then went to the pharmacy either the same day, or a day or two later, to collect it. This usually went like clockwork, with no issues. However, some participants talked about instances when the pharmacy did not have their prescription in stock and needed to order it from elsewhere. One participant talked about having to change her pharmacy because her original one continually had this issue. | “I’d go in and find my prescription hadn’t been sent through by the GP, and I’d have to go and collect it, or I’d have to come back on another day because they didn’t have it. I actually changed the pharmacy and once I changed the pharmacy it became, they didn’t seem to have as many issues as the original pharmacy did, so I stuck with them.” Interview 1  “Well, there has been occasions where there have been shortages of supply, not serious, but sometimes they have to give me a few pills and they get the rest in, one or two days later.” Interview 2 |
| One participant talked about her pharmacy providing the service of sending text reminders prompting her to fill in the prescription. | “Yeah, well, Boots just text me. Yeah, they do my resupply so, yeah. You just opt to do that.” Interview 7 |
| Payment |  |
| Having to pay for the treatment is an issue with worldwide resonance, as previously discussed. When asked about this, participants showed sympathy and were thankful for not having to pay for the treatment in the UK. | “I’d hate it if I’d had to pay for it. Yeah, I’d have to, but it’s expensive. Thank goodness that I, that we have got the NHS.” Interview 5  “Obviously most places it isn’t, no. I understand that. Oh, I would think so. Some people just couldn’t afford to do it.” Interview 13 |
| When asked whether they were told about their medication being free of charge, participants’ experiences varied. One mentioned being told about it in advance. | “I was told it was free because they gave me an exemption card almost straight away actually, which I felt very blessed in having that.” Interview 6 |
| However, some participants were not told their medication was free and found out later by themselves. One participant had to pay for a couple of months before finding out about the process of applying for free medication. | “Yeah, I didn’t know that I could get this as, I didn’t, I thought I had to pay for my prescriptions initially and that wasn’t, nobody told me that I could have them free. I found that out later but it was quite soon afterwards, but initially I wasn’t told that.” Interview 1  “No, I paid for it, just for a couple of months, until somebody said you have to fill in this form. In this country, if you fill in a form, cancer drugs are free, but they don’t tell you that. My oncologist didn’t tell me that. I had to find, I was told by somebody else, then filled in the form and got my cancer drugs free, but they don’t tell you.” Interview 4 |
| One participant reported her experience with health insurance. She talked about the support her health insurance company provided her throughout the treatment and how cooperative they were when she needed to do further tests and examinations. | “Yeah, well I’d have to say I was in the Irish system, Republic of Ireland, before I moved here, and private health insurance is normal. My husband is American, again private insurance is the norm. So when I came, started back here, and I’d been a student here actually − but anyway, when I came back in 2011, I took out BUPA health insurance, so I had private insurance. And I have to say the treatment was brilliant. So the GP, I would say, is my main go-to person for anxieties and that kind of thing. But BUPA, but if I need any further support, BUPA has always provided that.” Interview 4 |
| Cancer and society |  |
| According to one participant with expertise, cultural differences play a “huge” part in breast cancer, women from different cultures dealing with cancer differently. | “Yeah, of course, yeah. And also you’ve got cultural differences as well which we find, that’s one of the problems in the UK is that people from ethnic backgrounds quite often they won’t talk to anybody about, well, about breast cancer anyway and then, and certainly not been given information about being able to help themselves. Yeah, huge.” Interview 13 |
| How cancer is depicted in a society was another factor discussed in the interviews. One participant made clear her preference for keeping her own cancer diagnosis a secret, while another talked about how cancer is still a taboo topic, meaning that talking about it with strangers could be considered difficult for some survivors. | “The only thing I was, the only time is, you’re having it, that’s fine, you’re getting on with it, then you lose your hair and then you know that everyone knows you’ve got it, and that’s, I’d rather people didn’t know. Not because I’m embarrassed, but just because I didn’t need them to know. Do you know what I’m saying?” Interview 5  “And some people still fear cancer. The taboo’s being broken down but there is still, I don’t know if there is in your country, but here there still is a taboo about, a bit of a taboo about cancer, not as much as it used to be.” Interview 14 |
| The end of the treatment |  |
| Participants are prescribed hormone therapy for five years and told that at the end of that time they would need to come back to have their prognosis reassessed and discuss whether they would benefit from extending the treatment for a further five. Ultimately, reaching the end of the treatment and deciding whether to stop the medication was shown to be a dilemma in itself. Most women found the decision difficult, some deciding to continue treatment, others to discontinue it – not without fearing the possibility of recurrence. | “So, they said at the beginning, five years. When I went back, they’ve said that the history, new evidence has shown that patients that stay on it for ten years is more successful with the cancer not returning. So, I think they, for the price I think they just keep you on it for the ten years, and I assume you just stop after ten years.” Interview 3  “As to whether I need to stay on it any longer or whether they’ll terminate it after five years.” Interview 6 |
| Participants’ reaction to the prospect of being on the treatment for ten years rather than five years varied. Some were happy to continue if it made a difference and reduced the chances of any recurrence. Others were more reluctant, either accepting reluctantly or refusing to continue. Some of those who had not yet reached that stage of the treatment expressed a desire to terminate the process. | “Now if they can prove to me that, that won't make any difference, I would probably go along with it because statistics say, whereas if they say there’s another drug that I can take for the rest of my life that would stop cancer coming back, I would probably take that as well if, because it’s a thing you don’t want.” Interview 12  “Some of them can’t wait to get off it, but most people, I have to say that most people, when they were told that they could take it for ten years, they chose to do that. They got to the end of their five and they were, right, OK, well, then I’ll, that’s the end of it or if they’d perhaps for whatever reason they’d said, no, I’ll go for ten.” Interview 13  “Oh, once I got to the end, my last, yes, because then there was new research out and what they wanted me to do was take it for another two years and I just said ‘no’.” Interview 14  “As to whether I need to stay on it any longer or whether they’ll terminate it after five years. I’d like it to be terminated.” Interview 6 |
| The experience of reaching the end of the treatment also differed from one participant to another. While some participants were happy to reach the end of the line and quite excited to stop the treatment, others were worried that stopping the treatment would mean losing its protective properties. | “Relieved. I just hope that it hasn’t damaged my joints and that when you stop taking them, that the pain and everything will go and the hormone still might kick in and I might get nicer hair, and things that I had before might come back.” Interview 3  “Yeah, they told me, yeah. If they’d have said to me, you’ve got to be on it on the rest of your life, even though obviously it gives me the hot flushes, I still would have done that, yeah.” Interview 5  “Well, I just think if you stop taking it after ten years, how do you not know, that’s what worries me, because I think if then the oestrogen’s produced, I don't understand why you don’t take it for life.” Interview 10  “No I don’t think so, I think, I go by advice really. It will be scary coming off tablets, but equally it’ll be quite nice not having to take them.” Interview 12 |
| When given the choice to stop, some participants decided to stay on the treatment. One participant mentioned being given the option to stop by her healthcare provider after finishing the five-year course, but decided to continue treatment for an extra two, eventually stopping after seven years on the treatment, more than the recommended duration at the time. Another participant was still taking the treatment 16 years after her initial prescription. This is quite similar to another participant, who, as seen below took a similar decision. | “I was asked after five years if I could, if I would like to come off it, but we are very nervous as ladies when we’ve had breast cancer and I elected to stay on it for a couple more years, even though I did have some side-effects.” Interview 13  “They didn’t tell me at the time and I’m still taking it now and I find it very reassuring that I’m still taking it now, and I’ve got a very sympathetic GP, who is still prescribing it for me.” Interview 2  “Another who has bald patches anyway and wears a wig full-time and she’s had a mastectomy, she has persuaded her doctor that she’s so worried about recurrence that she wants to remain on it the rest of her life. She’s a little bit older than me but she wants to be on it.” Interview 9 |
| One participant talked about the fact that she was not told when she reached the end of the treatment and how she needed to ask about it herself, which made her feel abandoned and forgotten. Another woman shared a similar experience and decided to go with it and not stop the treatment. | “I don’t think that was handled very well because I was thinking, well if I hadn’t have initiated this conversation would I have just continued to take the drug? But it was because I knew at the outset that it was for a period of time that I asked about it. Nobody brought it up with me, and I think that’s a concern because you do feel forgotten about then.” Interview 1  “They didn’t tell me at the time and I’m still taking it now and I find it very reassuring that I’m still taking it now, and I’ve got a very sympathetic GP, who is still prescribing it for me.” Interview 3 |
| Another concern some women expressed was whether their healthcare provider’s suggestion for them to stop the treatment was actually in their best interest or might have been made due to financial concerns on the part of the healthcare system. | “I suppose that was a more critical point. Whether or not it was beneficial to have continued with it or to stop it, and was I, was the decision to stop it, which was taken by the professionals because they’d obviously decided to prescribe it for this period of time, was that based on the right criteria, for the right reasons, in the patient’s best interest? Or was there some financial constraints, because at the time I knew I was taking quite an expensive drug, when it first started, but I had, knew that I had access to the best choices at the time, so yeah, that was fine.” Interview 1  “I did feel a little bit well. I wonder if this is just a cost thing for the NHS. Whether it’s about cost rather than it truly being only a, there’s only a one percent benefit so it’s negligible.” Interview 11 |

**Table S3. Themes, their explanations and example quotes relating to the third category:** ***Taking a chance: stopping the treatment early***

| Severity of side-effects and poor quality of life | Example quotes |
| --- | --- |
| One participant talked about how the treatment affected her life negatively, making her feel tired and in constant pain, until she felt she was hardly living at all. She felt she was taking the treatment to merely “exist”, not to “live”. | “And I think that’s an important thing, you're, for me it was an existence, it was not living at all.” Interview 14 |
| Despite her dedication and commitment to taking the treatment for the original five years, when recommended to continue with it for longer, she refused. | “Oh, once I got to the end, my last, yes, because then there was new research out and what they wanted me to do was take it for another two years and I just said ‘no’.” Interview 14 |
| The treatment was crippling, and it was not just her quality of life that was affected. She felt the treatment impacted every aspect of her life. The treatment and its side-effects became her sole focus and by the end consumed her entirely. | “It’s joining up the dots, when it’s crippling, when it’s crippling and it’s for a number of years − we’re not talking about a few months here − out of your life, you give up your life when you're having something like this, to a certain extent, well to a big extent for me, actually.” Interview 14 |
| This participant felt that had the side-effects not been so bad, she might have continued on the treatment longer. But by the end, the cumulative effects of breast cancer, hormone therapy and the continuous infections made her decide to stop. The treatment interfered with her life so much that she reached her breaking point, making her feel she had no option but to stop. | “Well when it came, had the experience of taking it not been so bad, I probably would have gone on for the next few years. Having discussed that with the people in the osteoporosis clinic as well, can I go on having the intravenous zoledronate and things like that, I probably would have thought OK, if it wasn’t interfering with my life, but the effect on my life, the cumulative effect of everything, I was so weak when I started the cancer treatment and I just, I, you could have knocked me down with a feather, it was hell, it was hell and I just thought, ‘no’.” Interview 14 |
| This participant questioned the point of continuing on treatment. She felt that had she been younger, she might have persisted with the treatment longer, but took the decision to stop as she had reached the stage of valuing quality of life over longevity. This reasoning is quite similar to that of another interviewee, who thought that had she been older she might have decided against taking the treatment. | “What’s the point? This was, the chemo was hell, and then another five years of hell, am I going to have another two years of hell, that’s over seven years. What’s the point of this treatment, why was I treated for breast cancer in the first place? To have a life. Maybe if I was a lot younger, I would have thought maybe, maybe that’ll increase my chances. I was 61 when I was diagnosed. I'm not going to live forever, I would like to have some quality of life. So, I just thought, ‘no’.” Interview 14  “I do agree with that. I think if I was older I definitely wouldn’t take it, definitely no.” Interview 3 |
| Another participant also mentioned some people start the treatment for a while, suffer from the side-effects, then decide to stop. | “Yeah, some people, yeah, some people will take it for a while and hope that they might, it might go away and then when it doesn’t they then say, no, do you know what, I’m not going to take it anymore. I think that it’s, I think it’s, that again is a matter of choice.” Interview 13 |
| Stopping the treatment on advice |  |
| One more reason mentioned in the interviews for stopping the treatment was being given the choice to stop by the healthcare provider. | “I have another friend, an older colleague who works at the [….] and she stopped taking it because she was advised to.” Interview 9 |
| Another participant was similarly given the choice by her healthcare provider to either continue on the treatment or stop it after the initial five years. Her healthcare provider felt that despite the benefits of continuing on the treatment, they were outweighed by the side-effects. | “When it came to my five years, end of my five years, they had got the benefit of that study and when I spoke to my consultant about, I understand that if you take it for ten years then you’re, there is a benefit to that, he told me that the benefit was only about one percent, so it wasn’t worth the, it wasn’t worth taking it for ten years for a one percent benefit of not getting breast cancer, balanced with bones crumbling.” Interview 11 |
| When asked whether participants might decide themselves to stop the treatment, without discussing their decision with anyone, a participant working for a cancer support group thought this was a possibility. | “Yeah, sometimes they do it on their own. They don’t take, talk to anybody else.” Interview 13 |
| Changing priorities |  |
| Another reason for ceasing treatment was participants’ changing priorities while taking the medication. When asked whether she might consider stopping the treatment prematurely, one woman talked about the possibility of doing so if, down the line, she decided to have another child. To her, having another child would in that eventuality have taken priority over continuing her treatment. | “Yes, if I was about to have another child. But if not, then no, I don’t think so. I normally comply with what they tell me to do, to be honest, because other people they have more evidence on how to treat stuff, so I say, OK fine, I’m not an expert in this area so I follow instructions.” Interview 8 |
| Lack of trust |  |
| One participant who survived breast cancer and was now involved in supporting others, talked about how some women might have issues with prescribed medicines in general. These women might decide to stop their treatment due to a lack of trust in it and/or in the medical system itself, or simply be due to their fear of the potential side-effects. | You do get the odd ones who don’t actually want to take them at all because, and they usually have some sort of problem with prescribed medicines overall. But I’ve come across that as well, where people don’t want to take tablets.” Interview 13  “Yeah, I think it’s just if someone has got a real block about taking prescribed medicines, putting stuff − as they see it, poison − into their bodies, they really don’t want to do that. And other people, the reason they don’t want to take it is because they’re getting bad side-effects.” Interview 13 |
| Taking a chance by stopping treatment |  |
| One participant also talked about a family friend who decided not to undergo the treatment and instead to ‘take her chances with cancer’. | “Like my mum’s friend that had breast cancer and decided that she didn’t want any treatment, she didn’t, she just, so I do actually, through my own experiences, know that this happens.” Interview 11 |
| Benefiting from stopping treatment |  |
| Stopping the treatment and regaining a sense of normalcy in one participant’s life and made them feel “psychologically brilliant”. Although stopping the treatment did not have a sudden physical effect, the participant nevertheless reported feeling much better. Physically, it took the participant four years after stopping the treatment to ‘feel her old self’. | “Psychologically brilliant; physically there was no sudden change.” Interview 14  “Yes, after the, so October 2015 I stopped the letrozole and where are we now? October 2019. It was only earlier this year that I really felt it had finally, because even a slight thing would trigger a headache and my viral infection, everything.” Interview 14 |

**Table S4. Themes, their explanations and example quotes relating to the core category: *Hobson’s choice or a horned dilemma***

| Hobson’s choice | Example quotes |
| --- | --- |
| The findings in this study, in essence, match those identified earlier. Therefore, some participants did not think they had a choice about whether to take their treatment, the decision being akin to Hobson’s choice, given that the alternative to taking the treatment was, effectively, “dying early”. | “Just that, that was the only choice I had, It is a strange feeling that, you feel like you just have to do what you’re told. But I don’t feel there was a choice for me.” Interview 6  “Yeah, you have to take it because otherwise, it's the otherwise, what would happen if you don't take it. So you have no other choice to be honest, yeah that's true.” Interview 8  “I haven’t thought of not taking it. So yeah.” Interview 10 |
| Horned dilemma | Example quotes |
| On the other hand, some participants struggled daily with the decision to continue or stop the treatment, akin to a horned dilemma. | “When you get out of bed in the morning and then you have trouble even starting to walk and it can take a couple of hours to get your joints going again, or it’s like, when I’m sitting in the office sometimes, and I absolutely feel like I’m going to combust because I’m so hot. And any minute now you think your whole body’s going to blow apart, but the thing is, is, you could stop the drugs and die. It’s weighing it up, isn’t it? And, I guess each one is different in what our priorities are.” Interview 6  “Yeah, yeah. There are some people that go, I’m going to fight it regardless, and there are others that go, you know what, I don’t care, I’m going to give up, like my partner did. And I can see this in respect of some of the ladies within the breast cancer group where some of them have gone, you know what, I don’t care anymore.” Interview 11 |
| Changing nature of the decision | Example quotes |
| However, there were two participants who took the decision to stop treatment after having taken it for five years. Their perception of the treatment had changed from a Hobson’s choice to a horned dilemma over time, resulting in discontinuation. | “Yeah, it is Hobsons’s choice really, I haven’t thought of not taking it. So yeah.” Interview 14  “Oh, once I got to the end, my last, yes, because then there was new research out and what they wanted me to do was take it for another two years and I just said ‘no’. “What’s the point? This was, the chemo was hell, and then another five years of hell, am I going to have another two years of hell, that’s over seven years. What’s the point of this treatment, why was I treated for breast cancer in the first place? To have a life. Maybe if I was a lot younger, I would have thought maybe, maybe that’ll increase my chances. I was 61 when I was diagnosed. I'm not going to live forever, I would like to have some quality of life. So, I just thought, ‘no’.” Interview 14 |
